# Evaluating performance and potential clinical benefit of the Swedish On Scene Injury Severity Prediction (OSISP) model for prehospital field triage on Norwegian trauma data

**DOI:** 10.1186/s13049-026-01662-w

**Published:** 2026-07-24

**Authors:** Anna Bakidou, Eva-Corina Caragounis, Magnus Andersson Hagiwara, Olav Røise, Anders Jonsson, Bengt Arne Sjöqvist, Stefan Candefjord

**Affiliations:** 1 Department of Electrical Engineering, Chalmers University of Technology, Gothenburg, SE-412 96 Sweden; 2 PreHospen - Centre for Prehospital Research, Faculty of Caring Science, Work Life and Social Welfare, University of Borås, Borås, SE-501 90 Sweden; 3 Department of Surgery, Institute of Clinical Sciences, Sahlgrenska Academy, University of Gothenburg, Gothenburg, 413 45 Sweden; 4 Department of Surgery, Sahlgrenska University Hospital, Region Västra Götaland, Gothenburg, 413 45 Sweden; 5 Research and Development Department, Division of Orthopedics, P.O. Box 4950, Nydalen, Oslo, Norway; 6 Institute of Clinical Medicine, Faculty of Medicine, University of Oslo, P.O. Box 1072, Blindern, Oslo, Norway; 7 Norwegian Trauma Registry, Division of Emergencies and Critical Care, Oslo University Hospital, Oslo, 0424 Norway

**Keywords:** Artificial Intelligence (AI), Diagnostic modelling, External validation, Machine Learning (ML), Norwegian Trauma Registry (NTR), On Scene Injury Severity Prediction (OSISP), Prehospital care, Swedish Trauma Registry (SweTrau), Trauma

## Abstract

**Background:**

Rule-based field triage protocols used by Emergency Medicine Service (EMS) personnel for assessment and prioritization of trauma patients achieve suboptimal field triage performance. An On Scene Injury Severity Prediction (OSISP) model was previously developed on Swedish trauma data with indications to improve field triage performance. This study aims to apply OSISP on external Norwegian data to assess its performance and clinical impact on unseen data.

**Methods:**

Adult trauma incidents involving EMS resources at the scene of incident were included. An eXtreme Gradient Boosting OSISP model was developed on Swedish trauma registry (SweTrau) data for 2013–2020 and trained to predict severely injured patients defined as new injury severity score (NISS) > 15. OSISP was evaluated on Norwegian trauma registry (NTR) data for 2017–2022. The model performance on NTR data was evaluated using overall performance measures for discrimination and calibration (receiver operating characteristic curve, precision-recall curve, area under these curves, Brier score, calibration curve, calibration slope, calibration in the large). OSISP’s clinical impact was assessed by evaluating its under- and overtriage rates and comparing to current clinical outcome and mortality.

**Results:**

The raw data from SweTrau and NTR had 75,602 and 76,529 registrations, with 47,357 and 29,709 remaining after applying eligibility criteria. In the included data, there were 16.9% and 25.2% patients with NISS > 15 in SweTrau and NTR, respectively. The OSISP predictors represented age, sex, details of the patient’s condition, incident and injury details, vital signs, and administrative details. OSISP applied on NTR data yielded an overall performance of AUC_ROC_=0.83, AUC_PR_=0.64, Brier score = 0.14, calibration slope = 0.79, and calibration in the large=–0.09. On its own, OSISP reduced undertriage to 5.0% but increased overtriage to 61.0%, while the complementary use (NTR’s triage outcomes combined with OSISP’s predictions) reduced undertriage to 2.7%, overtriage to 19.7% (compare to current clinical practice with an undertriage = 56.0% and overtriage = 30.6%), and mortality from 4.1% to 3.4–3.5%.

**Conclusions:**

OSISP’s overall performance was successfully validated and a potential capability to improve triage and decrease mortality was observed, also for elderly and patients from the northern region, groups often missed with current triage tools.

**Registration:**

Not applicable.

**Trial registration:**

Not applicable.

**Supplementary Information:**

The online version contains supplementary material available at 10.1186/s13049-026-01662-w.

## Background

The last decades have brought increased demand on prehospital care by Emergency Medical Services (EMS) [1–4], a result of several factors such as an aging population [2, 3], increased mental health issues [4], and a change in attitude regarding patient perceived urgency [5]. Trauma represents a large proportion of EMS assignments [6], it is estimated to cause 8% of deaths worldwide [7] and often results in lifelong disability [7, 8], leading to global cost estimates of US$1.9 trillion [8]. To increase chance of survival, international recommendations suggest that severely injured patients should be transported directly to care facilities with the highest levels of resources (trauma centers, TC) [9]. The field triage protocols used to assess a patient’s condition can be retrospectively evaluated using the metrics undertriage (severely injured patient transported to non-TC, NTC), and overtriage (not severely injured patient transported to TC) [9]. The American College of Surgeons Committee on Trauma (ACS-COT) recommends under- and overtriage levels of 5% and 25–35%, respectively [9]. In practice, however, the recommended levels are difficult to achieve [10, 11], with reports of suboptimal under- and overtriage rates ranging from 9–61% and 10–90%, respectively [12–15]. In particular, geriatric patients and patients in rural regions suffer from high proportions of undertriage [16, 17]. All in all, EMS personnel face a difficult task as there is a high pressure to handle an increasing volume of missions with less severe conditions – that are still complex and time-consuming [18] – while managing challenges with triaging, and optimizing care for critical conditions [19]. Development of technologies to support EMS personnel to assess trauma is therefore essential to ensure patient safety.

Norway and Sweden are two Nordic countries that share similarities, e.g., trauma patient cohorts [17] and geographical challenges, but have different organizations for managing trauma care. Norway has an established national trauma system with defined TCs and transportation guidelines for severely injured patients [17], while Sweden has 21 healthcare regions responsible for providing care within each region and there is no national trauma system [20]. In studies evaluating the trauma care, high proportions of under- and overtriage when compared with ACS-COT’s recommended levels have been reported in both countries [17, 20–22]. A national mortality rate of 4.0% and 3.1% have been reported in Sweden and Norway [17, 20], with an increased risk of mortality for severely injured in the North of Norway [17]. Meanwhile, a reduced mortality has been reported for severely injured when treated at TC compared with NTC [17, 20]. Improving the field triage tools to more accurately identify severely injured in need of direct transportation to TC therefore holds potential to reduce mortality.

Our research group *Care@Distance* (based at Chalmers University of Technology, Gothenburg, Sweden) and colleagues aim to develop clinical decision support systems (CDSS) to support EMS, especially for critical conditions such as trauma, and to use data fusion and Artificial Intelligence (AI) to optimize risk models. The previous work on trauma has developed the concept On Scene Injury Severity Prediction (OSISP), referring to a CDSS that can be deployed by EMS personnel on the scene of incident to assess the risk of a patient being severely injured. The OSISP concept has been tested in proof-of-concept studies with data representing motor vehicle crashes [23–25], as well as incidents registered in the Swedish Trauma Registry (SweTrau) [26], showing potential to reduce both under- and overtriage rates. Similar risk models are developed by van Heijl and associates in the Netherlands [27], with potential clinical benefits in line with OSISP’s.

Several studies indicate the potential to improve clinical outcomes with AI, however, clinical implementation is rarely achieved [28]. One obstacle is lack of model validation [28], especially external validation where models are evaluated on new data with a structural difference compared to the development data, e.g., data from different locations or care settings [29]. The observed change in performance during external validation may be used as an indicator if a model’s performance can be reproduced for similar but new data [29], which helps researchers avoid research waste and secure patient safety for future clinical implementation. The aim of this paper was to study how OSISP performs on future patients by conducting an external validation, where OSISP models developed on Swedish trauma data are applied and evaluated on Norwegian trauma data, and evaluate potential clinical benefits for improving triage and recognize vulnerable patient groups that are missed in present clinical routine.

## Methods

### Study design

This is a registry study that externally validated AI-based OSISP models on Norwegian trauma data to evaluate if the predictive ability is transferable to a similar trauma population, which represented unseen data for the developed algorithms. The study followed the Transparent Reporting of a multivariable prediction model for Individual Prognosis Or Diagnosis (TRIPOD) + AI [30] for reporting and an additional file presents the checklist [Additional file 1]. No protocol was prepared.

### Setting and sources of data

The OSISP model utilized in this study was developed in a previous study using data from Sweden, an oblong country with a land area of about 410,000 km^2^ [31] and a population of about 25.8 people/km^2^ [32]. There are 21 healthcare regions, each responsible for its own trauma care, with regional organizations and regional TCs. The TC levels defined by ASC-COT are not transferable to Swedish TC for several reasons: Sweden does not have enough trauma incidents to reach the minimum criteria for level I TC; all regional TC do not have the required resources for a level I TC (e.g., burn centers); and certain training and research outputs are not fulfilled (e.g., there are no trauma registrars nor surgical intensive care units). In this study, a TC in Sweden was therefore defined as being a university hospital and regional TC while a NTC was defined as a non-university hospital/non-regional TC that can admit and stabilize patients and send them to TC if need be, similar to the method in [20, 26]. Sweden has ten anaesthesiologist-staffed Helicopter Emergency Medical Services (HEMS) [33], with a varied accessibility across the regions and a few regions not being covered. There are efforts to unify the trauma care with a two-level national trauma alert criteria that follows a rule-based logic to assess if a patient has life-threatening injuries (level 1) or severe injuries (level 2) using anatomical and physiological criteria, injury mechanisms and additional observation points [34]. The findings collected during field triage of a patient are reported to the Emergency Department (ED) nurse, who uses the information to decide if a full or limited trauma team activation is needed at the receiving hospital [34].

In Sweden, data on major trauma incidents are collected in the national quality registry SweTrau [26], which started data collection in 2011 [35] based on the variable setup recommended in the Utstein protocol for uniform reporting of major trauma [36]. Registration is performed at each connected hospital by a designated nurse who summarizes information collected during the prehospital and in-hospital care phases [35]. There are in total 49 acute care hospitals and all have been connected to the registry since May 2022 [35]. At the time period represented in the model development data (year 2013 to 2020), 84–96% acute care hospitals were connected to SweTrau, of which 69–86% reported data [35]. The estimated coverage of severely injured ranged from 63 to 68% [35].

In this study, the OSISP model was externally validated on Norwegian trauma data. The rationale for targeting Norway were the similarities and differences with Sweden. Norway has a similar geographical shape with a land area of about 323,000 km^2^ [37], a population of about 17.0 people/km^2^ [38], 13 anaesthesiologist-staffed HEMS operating from 12 locations, and six anaesthesiologist-staffed rescue helicopters [39]. There are four trauma regions with one designated TC in each region, and 34 additional trauma hospitals admitting potentially life-threatening patients [39]. Seldomly, trauma cases might be admitted in ordinary surgical facilities and then transported to one of the 38 trauma receiving hospitals. The TC levels defined by ASC-COT are transferable to the Norwegian TC, with the TC in the southeast trauma region of Norway being a level I TC and the TC in the three remaining trauma regions being level II. The field triage protocol to assess a patient uses anatomical and physiological categories, injury mechanisms and additional observation points [40]. Findings during field triage are reported to the ED nurse coordinator via the dispatch unit, and based on the information a decision about trauma team activation is made [40]. In a recent study, good adherence to trauma criteria have been reported [41]. The shared geographical and demographical challenges combined with the structural difference of an established national trauma care system motivated an external validation on Norwegian trauma data.

In Norway, the equivalent of SweTrau is the Norwegian Trauma Registry (NTR), a national quality registry that also collects data based on the variable setup from the Utstein protocol [39]. Data collection started in 2015 and all the 38 trauma hospitals are obliged to deliver data to NTR [39]. At the time period represented in the external validation data (year 2015 to 2022), 85–100% of the hospitals reported to NTR, and there was 100% coverage for patients received with a trauma team and 92% coverage when patients not received with a trauma team was included in the coverage number [42]. The sample size needed from NTR was about 16,500, estimated with the sample size equations presented by Buderer in [43] by setting the sensitivity = 90% (equal to 10% undertriage), the specificity = 75% (equal to 25% overtriage), the significance level α = 0.05, the 95% confidence interval tolerance W = ± 1%, and the prevalence = 21% (estimated from NTR’s annual reports). SweTrau’s and NTR’s inclusion and exclusion criteria are listed in Table 1 [35, 42].

**Table 1:** Registry inclusion and exclusion criteria

	SweTrau	NTR
**Inclusion criteria**		
Traumatic event where a trauma alert has been activated	x	x
Traumatic event due to penetrating injury to head, neck, torso and extremities above elbow and knee	x	x
Patients with NISS > 15 (including transfers ≤ 7 days) regardless of trauma team activation	x	
New Injury Severity Score (NISS) > 12		x
Patients with a single head injury Abbreviated Injury Scale (AIS) ≥ 3		x
Prehospital deaths due to traumatic events		x
**Exclusion criteria**		
The only traumatic injury is a chronic subdural hematoma	x	x
A trauma alert is triggered without an underlying traumatic event	x	x
Drowning, inhalation injury, asphyxia injury (hanging/suffocation), where no other traumatic injury is involved.	x	x

SweTrau = Swedish Trauma Registry, used for development. NTR = Norwegian Trauma Registry, used for external validation. Translation to English and matching of criteria were conducted by authors A.B., E-C.C., and O.R

### Analytical workflow

**Fig. 1:**
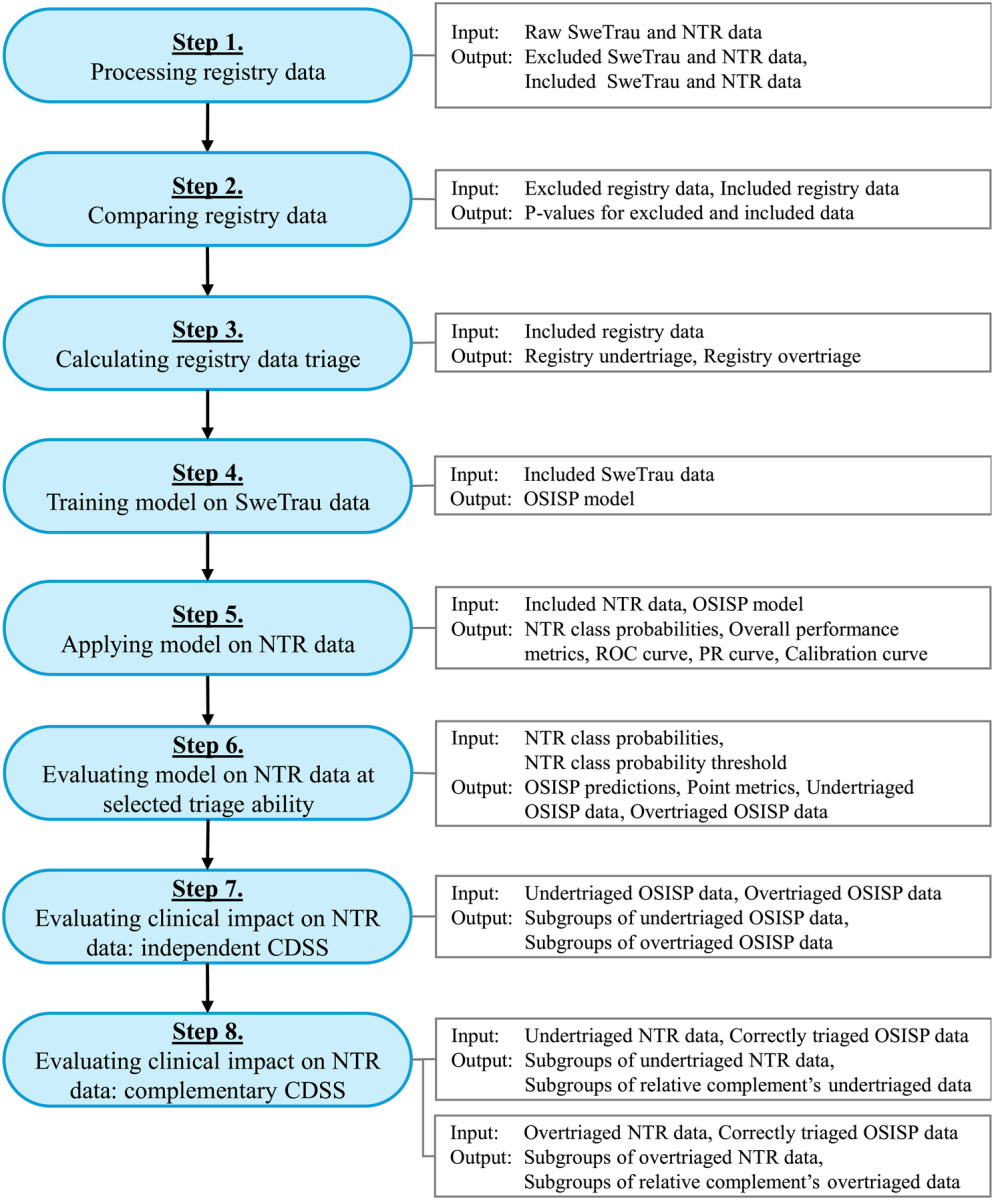
Analytical workflow

The analytical workflow consisted of the eight steps described below. The workflow is summarized in Fig. 1, accompanied with the input and output of each step.

#### Step 1. Data processing

Raw registry data were processed and prepared for analysis according to the procedure described in the development study [26]. In summary, the processing included variable generation (predictors and outcomes), data cleaning (managing missing data, exclusion of values not aligned with the registry manual for variables of interest to the study, and exclusion of duplications), application of eligibility criteria, and conversion of categorical values in predictors and outcomes to binary columns (i.e., one hot encoding) to enable a data format that is processable by the model. The processing was conducted for both SweTrau and NTR data, resulting in excluded and included dataset of each registry.

Generation of predictors were based on the identified predictors in the development study [26]: age, airway management, AIS region (head, face, neck, thorax, abdomen, spine, upper extremity, lower extremity, and external), cardiac arrest, dominating type of injury, motor component of Glasgow Coma Scale, sex, intention of injury, mechanism of injury, respiratory rate, response time, season of year and systolic blood pressure. All predictors were deemed collectable by EMS personnel at the incident scene and details of the predictor categorization are given in the development study. Because predictors originate from the Utstein protocol [36] and the AIS coding scheme [44], only two actions were needed to harmonize the SweTrau and NTR predictors: (1) the predictor level ‘*Unknown*’ was added to NTR predictors with no missing data, and (2) the NTR value “-1” corresponding to a not applicable status (e.g., that the hospital did not record that variable at the time) was coded as ‘*Unregistered*’ and excluded.

Generation of outcomes were based on the binary outcome that OSISP predicted in the development study [26]: if a patient was severely injured or not. The primary definition of a severely injured patient was a New Injury Severity Score (NISS) [45] > 15. A sensitivity analysis of secondary definitions was conducted using common NISS and Injury Severity Score (ISS) [45] thresholds to define severely injured patients [46]: ISS > 12, ISS > 15, and NISS > 12. ISS and NISS were considered appropriate outcomes as these are calculated based on AIS codes, which are systematically registered by trained health personnel professionals. The AIS codes, NISS scores and ISS scores were registered in both SweTrau and NTR, coded by the registry nurse at each hospital using information from the patient record [35, 47].

Missing and unknown data were grouped and added as an ‘*Unknown*’ predictor level to adapt the model to the prehospital context where complete data may not be available, according to the procedure in the development study [26]. No other missing data management technique was conducted as no notable differences in model performance were found when comparing different techniques in the development study [26].

Four inclusion criteria were applied for the selection of participants to represent OSISP’s intended environment: (1) involvement of prehospital resources; (2) direct transportation from the incident scene to the hospital; (3) adult trauma (≥ 15 years of age); and (4) alive at the scene. The study focused on adults as they are at a high risk of trauma [8], and also because paediatric trauma patients have a different physiological response to trauma [48], motivating development of a separate paediatric OSISP model in the future. Lastly, data from the initial period of NTR (1 January 2015 to 31 December 2016) were excluded due to inconsistencies in the registration process.

#### Step 2. Comparing registry data

To explore if there were any statistically significant differences between the excluded SweTrau and NTR data, as well as between the included SweTrau and NTR data, a test of independence were conducted for predictors and outcomes using Chi-square test, Fisher exact test and, in case of large contingency tables during the Fisher exact test, Monte-Carlo simulations [49]. For the excluded datasets, ‘*Unregistered*’ values were excluded during the test of independence due to being NTR specific and not relevant for the predictors or outcomes. The test of independence was considered statistically significant for p-values ≤ 0.05.

#### Step 3. Calculating registry data triage

Under- and overtriage rates were calculated with two different methods for the included SweTrau and NTR data: (1) equations based on ACS-COT definitions, and (2) equations based on trauma team activation (TTA) definitions. The first method used ACS-COT’s field triage definitions for undertriage and overtriage [9], where undertriage was defined as “*Severely injured patients transported to lower-level TC or other acute care facilities*” and overtriage was defined as “*Minimally injured patients transported to higher-level TC*”. The ACS-COT definitions are based on their TC classification of acute care hospitals, where a hospital can be classified as a NTC, or TC of level I, II or III [50]. If applying ACS-COT’s definitions of a TC (level I to III) on Swedish hospitals, none would fulfil the definition of a level I TC. In Norway, only Oslo University Hospital Ullevål meet the criteria for Level I TC [39]. In this study, approximations of ACS-COT’s TC definition were therefore used. In Sweden, university hospitals were approximated as TC and remaining acute care hospitals as NTC following the procedure of an earlier study by Candefjord and colleagues [20], resulting in seven TC. Note that Karolinska university hospital consists of two hospitals, one located at Stockholm and one at Huddinge. Only Karolinska at Stockholm should receive severely injured patients and was therefore coded as a TC while Huddinge hospital was coded as NTC. In Norway, trauma referral centres were classified as TC and remaining as NTC [39], resulting in four TCs. Neither SweTrau nor NTR collect data on the EMS personnel’s triage decision or the geographical location of the incident. The calculations, therefore, assumed that severely injured patients should have been transported to TC, and that not severely injured patients should have been transported to NTC. The undertriage (U) and overtriage (O) rates based on ACS-COT definitions were calculated with Eqs. 1 and 2, respectively, and encompassed the four different outcome definitions used in the sensitivity analysis.

  

1 $$\:{U}_{Registry}^{ACS-COT}=\frac{{N}_{ISS/NISS>12/15\:\&\:NTC}}{{N}_{ISS/NISS>12/15}}$$

  

2 $$\:{O}_{Registry}^{ACS-COT}=\frac{{N}_{ISS/NISS\le\:12/15\:\&\:TC}}{{N}_{ISS/NISS\le\:12/15}}$$

The second method to calculate under- and overtriage used TTA definitions of under- and overtriage [47], where undertriage was defined as “*Patients with very severe injuries (ISS ≥ 16) not received by a trauma team*”, and overtriage defined as “*Patients received by a trauma team without serious injuries and therefore theoretically do not need to be met by the trauma team*”. The TTA definitions consider patients being received by trauma teams rather than transport to TC. Like the approximations for ACS-COT’s calculations, it was approximated that severely injured patients should have been received by a trauma team and not severely injured not received by a trauma team. The undertriage (U) and overtriage (O) rates based on TTA definitions were calculated with Eqs. 3 and 4, respectively.

  

3 $$\:{U}_{Registry}^{TTA}=\frac{{N}_{ISS>15\:\&\:no\:TTA}}{{N}_{ISS>15}}$$

  

4 $$\:{O}_{Registry}^{TTA}=\frac{{N}_{TTA\:\&\:ISS\le\:15}}{{N}_{TTA}}$$

#### Step 4. Training model on SweTrau data

In the development study, OSISP was evaluated in a cross-validation setting and hold-out analysis [26], and no final model was saved. For this study, an OSISP model was therefore retrained using the included SweTrau data with the predictors and outcomes described in the development study [26]. The Extreme Gradient Boosting (XGBoost) model was selected as it performed well across the majority of metrics reported in the development study. The performance was verified to be similar as in the development study [26]. Four XGBoost models were trained in total, one for each outcome definition (ISS > 12, ISS > 15, NISS > 12, and NISS > 15).

#### Step 5. Applying model on NTR data

The retrained OSISP models were applied on the included NTR data and produced probabilities for registrations to be severely injured patients. The probabilities were used to visualize OSISP’s overall performance with a receiver operating characteristic (ROC) curve, precision-recall (PR) curve, and calibration curve. The ROC curve displays the sensitivity/recall against the false positive rate (1 - specificity), and a good model achieves a high sensitivity close to 1 and low false positive rate close to 0 [51]. The PR curve displays precision/positive predictive value (PPV) against sensitivity/recall, and a good model achieves both high precision and sensitivity close to 1 [52]. The calibration curve displays observed probabilities against predicted probabilities, and a good model achieves a close alignment, i.e., the curve is closely linked to the diagonal [53]. A model is underconfident if the calibration curve lies below the diagonal and overconfident if it lies above. The overall performance metrics calculated were area under the ROC curve (AUC_ROC_), area under the PR curve (AUC_PR_), calibration slope, calibration in the large and the Brier score. A good model achieves an AUC_ROC_ and AUC_PR_ close to 1 [51, 52]. The calibration slope and the calibration in the large are the estimated slope and interception of the calibration curve, and are close to 1 and 0 for good and bad models, respectively [53]. The Brier score sums the difference between the predicted probability and observed outcomes and divides the result with the number of predictions, and a good model achieves a low score close to 0 [54]. The analysis was repeated for each of the OSISP models. The OSISP model was not updated after the calibration analysis, and no class imbalance methods were applied.

The performance of the OSISP models in this paper were compared to published models and the clinical outcome in NTR. To the authors’ knowledge, the number of publications on predicting severely injured patients in the prehospital setting is limited. The comparison to published models was therefore limited to earlier OSISP publications [23–26], and studies related to the Dutch trauma model [27, 55].

#### Step 6. Evaluating model on NTR data at selected field triage ability

OSISP was evaluated on NTR data at two selected field triage abilities, 5% undertriage and 35% overtriage (the recommended triage levels by ACS-COT), to enable concrete interpretations of OSISP as a deployed model. When using ACS-COT’s field triage definitions, the sensitivity axis and the false positive rate axis of the ROC curve correspond to *1-Undertriage* and *Overtriage*, respectively. Because each data point on the ROC curve correspond to a probability threshold that converts larger probabilities to positive predictions (severely injured) and smaller probabilities to negative predictions (not severely injured), the ROC curve can be used to study a model with a fixed triage ability. In this study, the probability thresholds yielding a sensitivity of 0.95 and a false positive rate of 0.35 were used to study the OSISP models at the selected field triage abilities, hereafter referred to as OSISP-U5 (5% undertriage) and OSISP-O35 (35% overtriage). Each threshold was used to convert the produced probabilities to binary predictions and the point performance metrics accuracy, F1-score, PPV/precision, negative predictive value (NPV), Observed/Expected (O/E) ratio and sensitivity/recall were calculated as presented in Table 2 [53, 56]. In this study, a true positive (TP) corresponds to a severely injured patient predicted as severely injured, a false positive (FP) to a not severely injured patient predicted as severely injured, a true negative (TN) to a not severely injured patient predicted as not severely injured, a false negative (FN) to a severely injured patient predicted as not severely injured. The analysis was repeated for each of the four OSISP models (ISS > 12, ISS > 15, NISS > 12, and NISS > 15).

**Table 2:** Calculations of point performance metrics at selected triage abilities

Point performance metric	Calculation
Accuracy	(TP + TN)/(TP + TN+FP + FN)
F1-score	(2×Precision×Recall)/(Precision+Recall)
PPV/Precision	TP/(TP + FP)
Negative predictive value (NPV)	TN/(TN + FN)
O/E ratio	N positive observations/N positive predictions
Sensitivity/Recall	TP/(TP + FN)

PPV = positive predictive value, O/E = Observed/Expected, TP = true positive, TN = true negative, FP = false positive, FN = false negative

For both OSISP-U5 and OSISP-O35, undertriage (U) and overtriage (O) were calculated according to the ACS-COT definitions for field triage (Eqs. 5 and 6) and encompassed the four different outcome definitions used in the sensitivity analysis. Due to the limitations of not recording the EMS personnel’s triage decision or the geographical location of the incident, it was assumed that a positive prediction would be transported to TC and a negative prediction to NTC. The calculations were repeated for both OSISP-U5 and OSISP-O35, as well as each of the four outcome definitions (ISS > 12, ISS > 15, NISS > 12, and NISS > 15).

  

5 $$\:{U}_{OSISP}^{ACS-COT}=\frac{{N}_{ISS/NISS>12/15\:\&\:Negative\:prediction}}{{N}_{ISS/NISS>12/15}}$$

  

6 $$\:{O}_{OSISP}^{ACS-COT}=\frac{{N}_{ISS/NISS\le\:12/15\:\&\:Positive\:prediction}}{{N}_{ISS/NISS\le\:12/15}}$$

#### Step 7. Evaluating clinical impact on NTR data: OSISP as independent CDSS

The clinical impact on future patients with OSISP as an independent CDSS was estimated by comparing under- and overtriage rates in the NTR data with under- and overtriage rates produced by OSISP-U5 and OSISP-O35. To gain insights into fairness and performance for vulnerable groups, the triage accuracy rates were broken down into the following subgroups: age [15 (Pediatric), 16–45 (Young adults), 46–60 (Middle aged), 61–75 (Old adults), > 75 (Geriatric)] [20], sex [Female, Male], and region [Central, Northern, Southeast, and Western] [17]. Furthermore, 30-day mortality was reported for the under- and overtriage cohorts. The analysis was repeated for each of the four outcome definitions (ISS > 12, ISS > 15, NISS > 12, and NISS > 15).

#### Step 8. Evaluating clinical impact on NTR data: OSISP as complementary CDSS

**Fig. 2:**
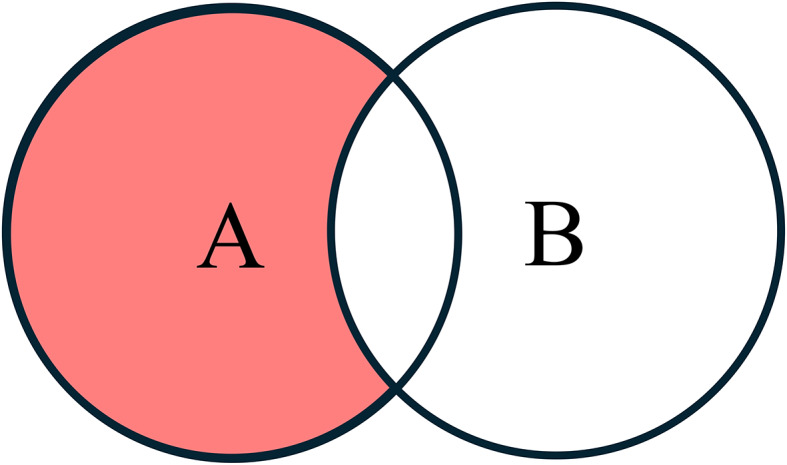
Venn diagram with relative complement marked. **A** = NTR’s incorrectly triaged patients (step 3), **B** = OSISP’s correctly triaged patients (step 6)

The clinical impact on future patients in terms of under- and overtriage was also estimated for OSISP as a complementary CDSS, meaning OSISP is used as a CDSS in combination with current triage tools. First, the incorrectly triaged patients in the NTR registry data (calculated in step 3) were used to approximate current triage tools’ performances. From these incorrectly triaged patients, patients correctly predicted by OSISP (calculated in step 6) were removed. The remaining proportion of incorrectly triaged patients was reported as the estimated clinical impact if using OSISP as a complementary CDSS, which may also be referred to as the relative complement (see the Venn diagram in Fig. 2). The analysis considered both the ACS-COT and TTA triage definitions (step 3) when calculating incorrectly triaged NTR data to approximate current triage tools’ performances. In the case of the ACS-COT definitions ($\:{U}_{NTR}^{ACS-COT}$ and $\:{O}_{NTR}^{ACS-COT}$), the analysis was repeated for OSISP-U5 and OSISP-O35, and repeated for each of the four outcome definitions (ISS > 12, ISS > 15, NISS > 12, and NISS > 15). In the case of TTA’s definitions ($\:{U}_{NTR}^{TTA}$ and $\:{O}_{NTR}^{TTA}$), the analysis was repeated for OSISP-U5 and OSISP-O35, but only for the outcome definition ISS > 15. The results were broken down as described in step 7, with the subgroups age, sex, and region. Furthermore, 30-day mortality was reported for the under- and overtriage cohorts.

A survival benefit analysis was conducted to estimate potential lives that could be saved by increasing the amount of severely injured treated at TC with improved triage accuracy, i.e., estimating the mortality reduction for benefitted severely injured patients incorrectly triaged by current triage protocols but correctly triaged by OSISP. Dehli et al. [17] found that severely injured patients in Norway had an odds ratio of 2.04 of surviving if admitted directly to TC compared to NTC. The number of potential lives to save was therefore estimated by assuming that the odds ratio corresponded to approximately 50% reduction in 30-day mortality. Based on the number of potential lives to save, mortality in the included data prior and posterior to the OSISP intervention were calculated. The analysis considered only ACS-COT triage definitions (step 3) when extracting the benefitted patients, with the triage definitions and analysis repetitions described above for the complementary clinical impact.

### Ethical considerations

In Sweden, the study was approved by the Swedish Ethical Review Authority and SweTrau. In Norway, the study was approved by NTR. No additional Norwegian ethical approval was needed since the work was approved by the Swedish Ethical Review Authority and conducted in Sweden.

### Software and availability of analytical code

The analysis was conducted using Python as programming language, with a modularly structured code with four main scripts generating the processed data, comparing the registry data, training the OSISP model, and evaluating the model. The code was executed at Chalmers Centre for Computational Science and Engineering. Microsoft Copilot (GPT-4), available through license provided by Chalmers University of Technology, was used as support during code debugging. The analysis code is not publicly available but can be reproduced using standard statistical software and methods described in the manuscript. The Python version and packages versions are presented in an additional file [see Additional file 2]. The OSISP model was saved in pickle format and may be provided by the authors on reasonable request.

## Results

### Participants and description of registry data

**Fig. 3:**
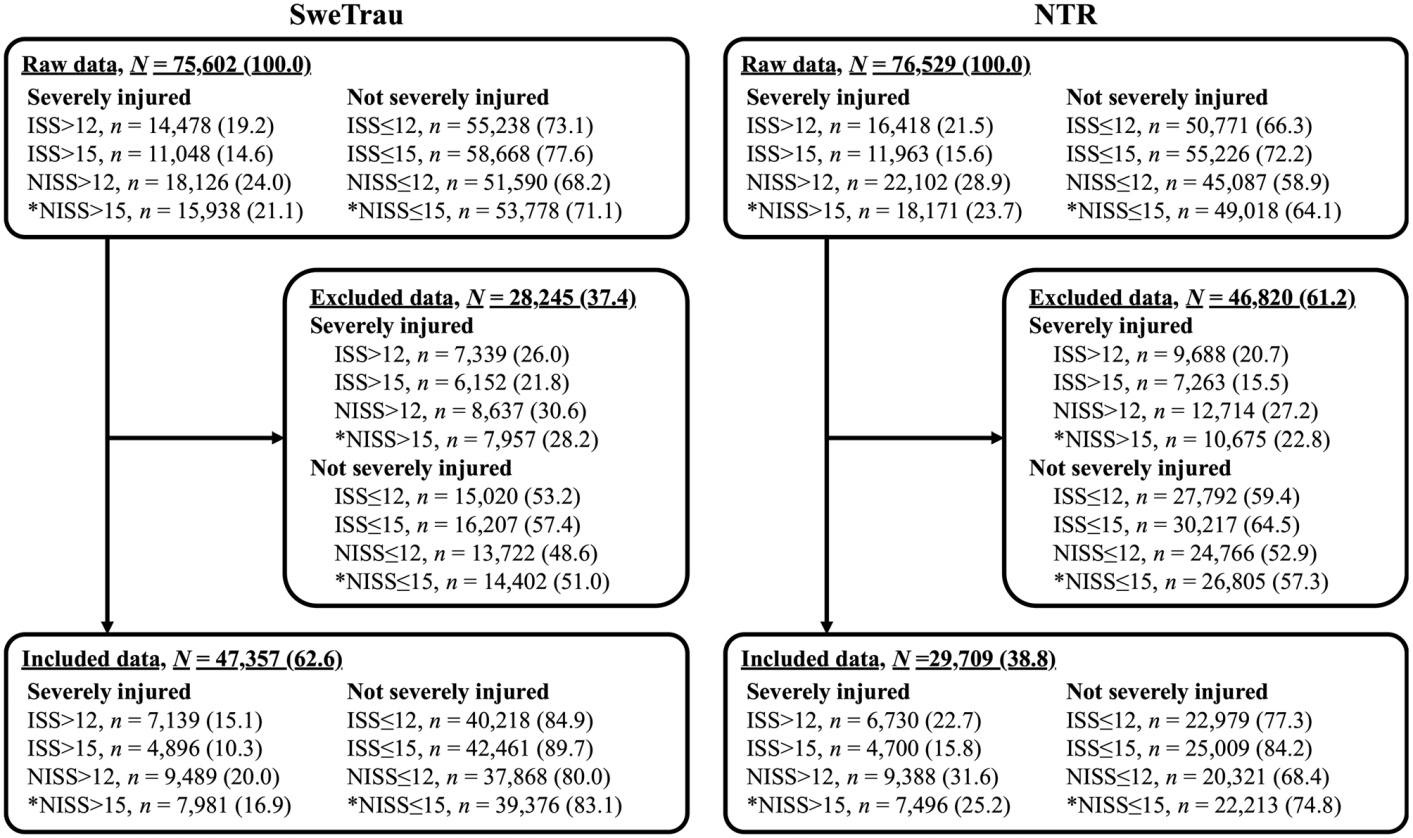
Flowchart of the patient selection. For each data, the distribution of severely injured and not severely injured, for each definition, are presented as counts and percentages in parenthesis. ISS = Injury Severity Score, NISS = New Injury Severity Score. *Primary outcome definition

Step 1 of the analytical workflow resulted in the flowchart of participant selection displayed in Fig. 3. The raw SweTrau data consisted of 75,602 registrations and after processing the data, 28,245 (37.4%) were excluded and 47,357 (62.6%) included. The raw NTR data consisted of 76,529 registrations and after processing the data, 46,820 (61.2%) were excluded and 29,709 (38.8%) were included.

Descriptive statistics of the outcome definitions for severely injured are included in Fig. 3. Descriptive statistics of the excluded and included NTR predictor data are presented in Table 3, and a corresponding table for the SweTrau data was reported in [26]. For all outcome definitions, the prevalence of severely injured were higher in the included NTR data compared to SweTrau.

**Table 3:** Distribution of predictor data in excluded and included NTR data

Predictor	Level	NTR Excluded [n (%)]	NTR Included [n (%)]
Age	≤ 55 (Young)	32,924 (70.3)	18,386 (61.9)
	> 55 (Elderly)	13,773 (29.4)	11,243 (37.8)
	Unknown	122 (0.3)	80 (0.3)
	Unregistered	1 (0.0)	0 (0.0)
Airway management	No	37,430 (79.9)	28,689 (96.6)
	Yes	838 (1.8)	912 (3.1)
	Unknown	7,535 (16.1)	108 (0.4)
	Unregistered	1,017 (2.2)	0 (0.0)
AIS region: abdomen	No	42,583 (91.0)	27,087 (91.2)
	Yes	4,237 (9.0)	2,622 (8.8)
AIS region: external	No	41,047 (87.7)	25,542 (86.0)
	Yes	5,773 (12.3)	4,167 (14.0)
AIS region: face	No	37,794 (80.7)	22,102 (74.4)
	Yes	9,026 (19.3)	7,607 (25.6)
AIS region: head	No	31,590 (67.5)	17,932 (60.4)
	Yes	15,230 (32.5)	11,777 (39.6)
AIS region: lower extremity	No	36,501 (78.0)	21,009 (70.7)
	Yes	10,319 (22.0)	8,700 (29.3)
AIS region: neck	No	45,782 (97.8)	28,723 (96.7)
	Yes	1,038 (2.2)	986 (3.3)
AIS region: spine	No	40,037 (85.5)	24,323 (81.9)
	Yes	6,783 (14.5)	5,386 (18.1)
AIS region: thorax	No	36,476 (77.9)	21,051 (70.9)
	Yes	10,344 (22.1)	8,658 (29.1)
AIS region: upper extremity	No	36,754 (78.5)	20,907 (70.4)
	Yes	10,066 (21.5)	8,802 (29.6)
Cardiac arrest	No	38,142 (81.5)	29,455 (99.1)
	Yes	222 (0.5)	160 (0.5)
	Unknown	7,439 (15.9)	94 (0.3)
	Unregistered	1,017 (2.2)	0 (0.0)
Dominating type of injury	Blunt	39,966 (85.4)	27,938 (94.0)
	Penetrating	1,717 (3.7)	1,657 (5.6)
	Unknown	4,541 (9.7)	114 (0.4)
	Unregistered	596 (1.3)	0 (0.0)
mGCS	1. No motor response	992 (2.1)	981 (3.3)
	2. Extension to pain (decerebrate)	143 (0.3)	139 (0.5)
	3. Flexion to pain (decorticate)	274 (0.6)	250 (0.8)
	4. Withdrawal from pain	564 (1.2)	585 (2.0)
	5. Localising pain	1,085 (2.3)	1,098 (3.7)
	6. Obeys commands/appropriate response to pain	27,349 (58.4)	23,544 (79.2)
	Unknown	15,396 (32.9)	3,112 (10.5)
	Unregistered	1,017 (2.2)	0 (0.0)
Intention of injury	Accident	38,910 (83.1)	26,339 (88.7)
	Assault	1,760 (3.8)	1,801 (6.1)
	Other	64 (0.1)	63 (0.2)
	Self-inflicted	1,167 (2.5)	1,117 (3.8)
	Unknown	4,718 (10.1)	389 (1.3)
	Unregistered	201 (0.4)	0 (0.0)
Mechanism of injury	Blunt object	3,858 (8.2)	2,511 (8.5)
	Explosion	143 (0.3)	97 (0.3)
	High energy fall > 3 m	9,290 (19.8)	6,308 (21.2)
	Low energy fall < 3 m	6,469 (13.8)	5,357 (18.0)
	Other	1,540 (3.3)	879 (3.0)
	Shot	149 (0.3)	144 (0.5)
	Stab	1,163 (2.5)	1,241 (4.2)
	Traffic - Bicycle injury	3,863 (8.3)	2,853 (9.6)
	Traffic - Motor vehicle injury	11,349 (24.2)	6,698 (22.5)
	Traffic - Motorcycle injury	2,549 (5.4)	2,117 (7.1)
	Traffic - Pedestrian	1,098 (2.3)	724 (2.4)
	Traffic - other	891 (1.9)	555 (1.9)
	Unknown	4,296 (9.2)	225 (0.8)
	Unregistered	162 (0.3)	0 (0.0)
Respiratory rate	0	56 (0.1)	59 (0.2)
	1–5	19 (0.0)	9 (0.0)
	6–9	78 (0.2)	65 (0.2)
	10–29	20,179 (43.1)	19,520 (65.7)
	> 29	1,817 (3.9)	1,611 (5.4)
	Unknown	24,671 (52.7)	8,445 (28.4)
Response time	< 8 min	6,310 (13.5)	5,832 (19.6)
	≥ 8 min	25,564 (54.6)	23,266 (78.3)
	Unknown	14,944 (31.9)	611 (2.1)
	Unregistered	2 (0.0)	0 (0.0)
Season of year	Autumn	11,281 (24.1)	7,012 (23.6)
	Spring	11,317 (24.2)	7,188 (24.2)
	Summer	14,342 (30.6)	9,143 (30.8)
	Unknown	0 (0.0)	0 (0.0)
	Winter	9,880 (21.1)	6,366 (21.4)
Sex	Female	15,435 (33.0)	9,457 (31.8)
	Male	30,416 (65.0)	20,252 (68.2)
	Unknown	0 (0.0)	0 (0.0)
	Unregistered	969 (2.1)	0 (0.0)
Systolic blood pressure	0	39 (0.1)	53 (0.2)
	1–49	11 (0.0)	6 (0.0)
	50–75	144 (0.3)	158 (0.5)
	76–89	325 (0.7)	360 (1.2)
	> 89	24,639 (52.6)	23,138 (77.9)
	Unknown	21,662 (46.3)	5,994 (20.2)

Distributions are presented by counts and percentage in parentheses. AIS = Abbreviated Injury Scale, mGCS = motor component of Glasgow Coma Scale

### Comparison of SweTrau and NTR data

The test of independence, conducted in step 2, to compare excluded SweTrau and NTR data marked all predictors but the AIS regions “*Lower extremity*” and “*Upper extremity*” as statistically different, and all outcomes were found statistically different. For the included SweTrau and NTR data, the test of independence marked all predictors but the AIS regions “*Abdomen*” and “*Upper Spine*” as statistically different, and all outcomes were found statistically different.

### SweTrau and NTR registry triage

Under- and overtriage in the SweTrau and NTR data, derived from step 3, are presented in Table 4. The triage definitions had a clear impact on the calculations, where the ACS-COT definitions resulted in larger undertriage values (39.4–58.1 versus 15.5–19.7) while the TTA definitions resulted in larger overtriage values (85.4–90.4 versus 30.3–44.4). For the ACS-COT definitions, NTR had the highest undertriage values while SweTrau had the highest overtriage values. For the TTA triage definitions, SweTrau had both the highest under- and overtriage values.

**Table 4:** Under- and overtriage rates in SweTrau and NTR registry data

Triage metric	Triage definition	Calculation	SweTrau [%]	NTR [%]
Undertriage	ACS-COT	Equation 1 with ISS > 12	42.5	56.6
		Equation 1 with ISS > 15	39.4	53.3
		Equation 1 with NISS > 12	43.2	58.1
		Equation 1 with NISS > 15	41.0	56.0
	TTA	Equation 3	19.7	15.5
Overtriage	ACS-COT	Equation 2 with ISS ≤ 12	44.1	31.2
		Equation 2 with ISS ≤ 15	44.4	31.6
		Equation 2 with NISS ≤ 12	43.4	30.3
		Equation 2 with NISS ≤ 15	43.5	30.6
	TTA	Equation 4	90.4	85.4

Under- and overtriage presented as percentages. ACS-COT = American College of Surgeons Committee on Trauma, TTA = Trauma Team Activation, ISS = Injury Severity Score, NISS = New Injury Severity Score

### OSISP’s performance on NTR data

**Fig. 4:**
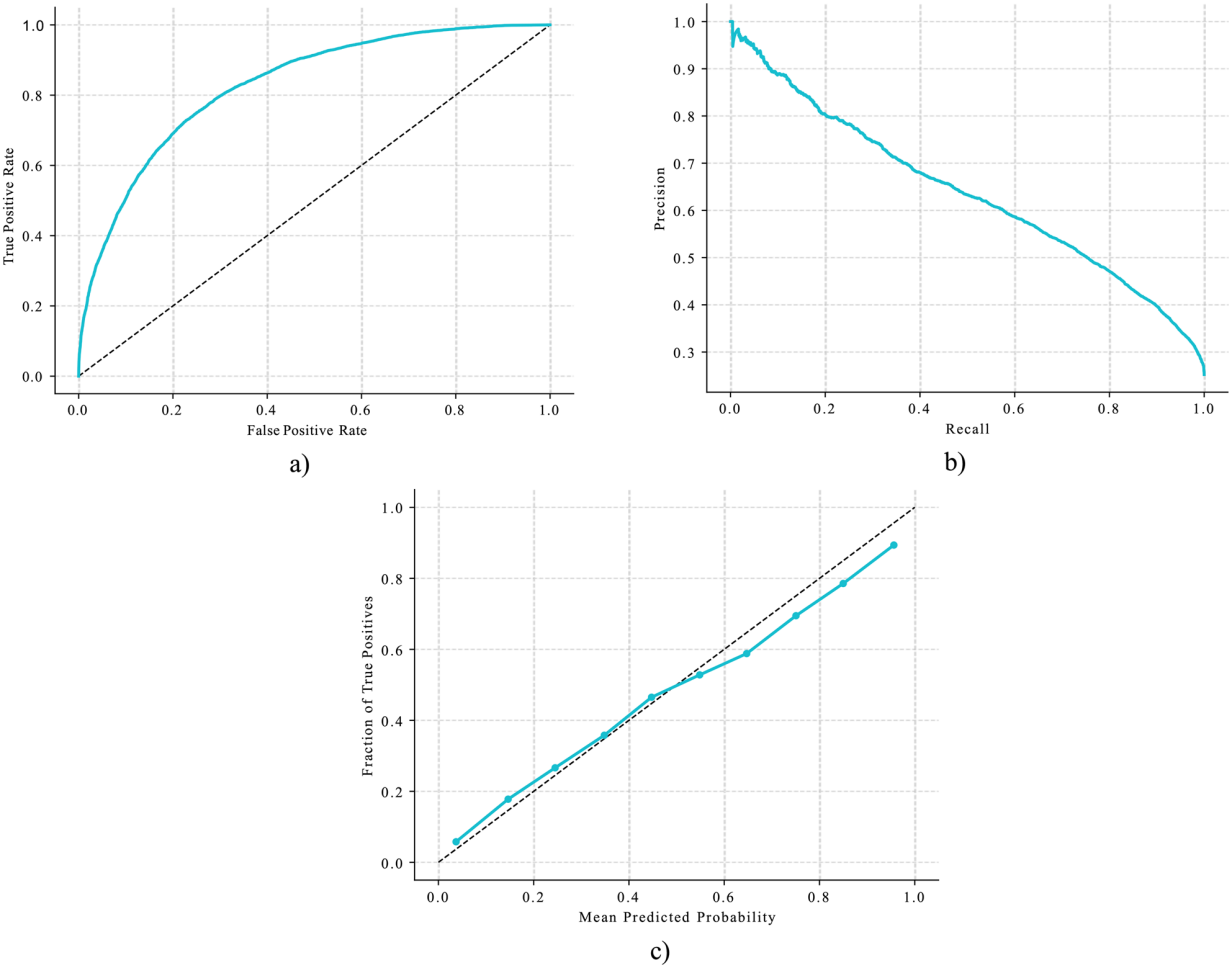
Overall performance of OSISP when trained to predict severely injured as NISS > 15. (**a**) ROC curve, (**b**) PR curve, (**c**) Calibration curve

Overall model performance metrics obtained from step 5 for OSISP when defining severely injured as NISS > 15 are reported in Table 5, and visualised in the ROC curve, PR curve and calibration curve in Fig. 4. The ROC curve reached toward the upper left corner while the PR curve had a close alignment with the diagonal, indicating similar model performance on the NTR data as in the SweTrau data. The AUC_ROC_ and AUC_PR_ showed that the OSISP model performed well in identifying severely injured in the NTR data. The calibration plot and metrics revealed a small miscalibration, causing OSISP to slightly underestimate patients at low risk of being severely injured, and to a moderate extent overestimate patients at high risk of being severely injured. This means that for patients that OSISP produced low probabilities of being severely injured, the actual risk in the registry data was slightly higher, while for patients that OSISP produced high probabilities of being severely injured, the actual risk in the registry data was lower. Corresponding results for the sensitivity analysis of alternative outcome definitions are presented in an additional file [see Additional file 3].

**Table 5:** OSISP overall performance when trained to predict severely injured as NISS > 15

Metric	Values
AUC_ROC_	0.83
AUC_PR_	0.64
Brier score	0.14
Calibration slope	0.79
Calibration in the large	–0.09

AUC_ROC_ = Area under the Receiver Operating Characteristics curve, AUC_PR_ = Area under the Precision-Recall curve

### OSISP’s performance on NTR data at selected triage ability

Based on the ROC curve data, OSISP was evaluated at ACS-COT’s recommended triage levels in step 6. Table 6 presents the model performance metrics for each evaluation point for the OSISP model trained to predict severely injured defined as NISS > 15. The OSISP-U5 prioritized a high recall (low undertriage), consequently increasing the number of false positives by a lot, which caused the model to perform rather modest for most of the performance metrics. The OSISP-O35 desired a moderate recall, consequently increasing the number of false positives to a lesser degree, which caused the model to perform more balanced for the remaining performance metrics. Corresponding results for the sensitivity analysis of alternative outcome definitions (ISS > 12, ISS > 15, and NISS > 12) are presented in an additional file [see Additional file 3].

**Table 6:** OSISP performance evaluated at ACS-COT’s recommended triage levels

Metric	OSISP-U5	OSISP-O35
Accuracy	0.53	0.70
F1-score	0.51	0.58
PPV	0.34	0.45
NPV	0.96	0.92
O/E ratio	0.36	0.54
Recall	0.95	0.83

PPV = Positive predictive value, NPV = Negative predictive value, O/E ratio = Observed/Expected ratio

### OSISP’s clinical impact on NTR data when used as an independent CDSS

In step 7, the under- and overtriage rates in the NTR registry data and the under- and overtriage achieved by OSISP as an independent CDSS are presented in Table 7, based on ACS-COT definitions for field triage. As expected, OSISP may improve either the under- or overtriage, but not both simultaneously. Notably, OSISP-U5 may substantially lower the undertriage from 56.0% to 5.0%, at the expense of an increased overtriage from 30.6% to 61.0%. The groups with the highest triage accuracy shared between the OSISP-U5 and OSISP-O35 were patients of 15 years of age, females, and the northern region. The groups with the lowest triage accuracy shared by the models were patients of 16–45 years of age, males, and the southeast region. Corresponding results for the sensitivity analysis of alternative outcome definitions are presented in an additional file [see Additional file 3].

**Table 7:** Under- and overtriage with OSISP (NISS > 15) as independent CDSS

	NTR^a^	OSISP-U5^b^	OSISP-O35^c^
	Undertriage; Overtriage	Undertriage; Overtriage	Undertriage; Overtriage
*Total[%]*	56.0; 30.6	5.0; 61.0	16.8; 35.0
*Age [%]*			
15	0.3; 0.5	0.1; 1.0	0.2; 0.4
16–45	15.0; 16.0	2.7; 26.2	7.9; 12.8
46–60	12.6; 6.3	1.1; 12.8	4.0; 7.5
61–75	14.8; 4.3	0.7; 11.2	2.5; 7.4
> 75	13.1; 3.5	0.4; 9.6	2.2; 6.7
Unknown	0.2; 0.0	0.0; 0.2	0.0; 0.1
*Sex [%]*			
Female	16.1; 9.8	2.0; 18.4	5.6; 10.0
Male	39.9; 20.8	3.0; 42.6	11.1; 25.0
*Region [%]*			
Central	4.3; 4.8	0.6; 8.4	1.9; 4.8
Northern	4.6; 1.8	0.4; 4.2	1.4; 2.4
Southeast	36.8; 17.4	3.3; 37.5	10.8; 21.6
Western	10.3; 6.5	0.7; 10.9	2.7; 6.2
*30-day mortality [% (n)]*			
Dead	11.4 (480); 1.7 (115)	11.5 (43); 2.1 (285)	7.5 (94); 2.7 (210)
Alive	88.6 (3,720); 98.3 (6,673)	88.5 (331); 97.9 (13,264)	92.5 (1,162); 97.3 (7,559)

Under- and overtriage rates presented as percentages and reported for (a) NTR based on ACS-COT definitions, (b) OSISP-U5 based on ACS-COT definitions, and (c) OSISP-O35 based on ACS-COT definitions. Mortality, reported as percentage of patients and number in parenthesis, is presented for the under- and overtriage cohorts separately, divided into the subgroups dead and alive that combined sums up to 100%

### OSISP’s clinical impact on NTR data when used as a complementary CDSS

In step 8, the under- and overtriage rates in the NTR registry data and the under- and overtriage made by OSISP as a complementary CDSS for ACS-COT definitions and TTA definitions are presented in Tables 8 and 9, respectively. Although both OSISP-U5 and OSISP-O35 as complementary CDSS improved both the under- and overtriage for the ACS-COT based calculations (Table 8), it is notable that OSISP-U5 achieved an under- and overtriage that fulfils the recommended ACS-COT levels. The groups with the highest triage accuracy shared between the OSISP-U5 and OSISP-O35 were patients of 15 years of age and females, while the groups with the lowest triage accuracy were patients of 16–45 years of age, males, and the southeast region. Corresponding results to Table 8 for the sensitivity analysis of alternative outcome definitions are presented in an additional file [see Additional file 3]. For the NTR based calculations (Table 9), the OSISP models also reduced the under- and overtriage. The groups with the highest triage accuracy shared between the models were patients of 15 years of age, females, and the northern region, while the groups with the lowest triage accuracy were patients of 16–45 years of age, males, and the southeast region.

**Table 8:** Under- and overtriage with OSISP (NISS > 15) as complementary CDSS for ACS-COT definitions

	NTR^a^	NTR&OSISP-U5^b^	NTR&OSISP-O35^c^
	Undertriage; Overtriage	Undertriage; Overtriage	Undertriage; Overtriage
*Total [%]*	56.0; 30.6	2.7; 19.7	9.8; 11.8
*Age [%]*			
15	0.3; 0.5	0.0; 0.3	0.1; 0.1
16–45	15.0; 16.0	1.3; 9.1	4.1; 4.7
46–60	12.6; 6.3	0.7; 4.1	2.3; 2.5
61–75	14.8; 4.3	0.4; 3.2	1.6; 2.3
> 75	13.1; 3.5	0.2; 3.0	1.7; 2.2
Unknown	0.2; 0.0	0.0; 0.0	0.0; 0.0
*Sex [%]*			
Female	16.1; 9.8	1.0; 5.9	3.3; 3.3
Male	39.9; 20.8	1.7; 13.8	6.5; 8.5
*Region [%]*			
Central	4.3; 4.8	0.3; 3.3	0.9; 1.9
Northern	4.6; 1.8	0.3; 1.2	1.0; 0.7
Southeast	36.8; 17.4	1.7; 11.3	6.6; 7.0
Western	10.3; 6.5	0.3; 3.9	1.4; 2.2
*30-day mortality [% (n)]*			
Dead	11.4 (480); 1.7 (115)	16.6 (33); 2.1 (93)	9.9 (73); 2.6 (68)
Alive	88.6 (3,720); 98.3 (6,673)	83.4 (166); 97.9 (4,287)	90.1 (663); 97.4 (2,553)

Under- and overtriage rates presented as percentages and reported for (a) NTR, (b) the relative complement with OSISP-U5, and (c) the relative complement with OSISP-O35. Mortality, reported as percentage of patients and number in parenthesis, is presented for the under- and overtriage cohorts separately, divided into the subgroups dead and alive that combined sums up to 100%

**Table 9:** Under- and overtriage with OSISP (NISS > 15) as complementary CDSS for TTA definitions

	NTR^a^	NTR&OSISP-U5^b^	NTR&OSISP-O35^c^
	Undertriage; Overtriage	Undertriage; Overtriage	Undertriage; Overtriage
*Total [%]*	15.5; 85.4	0.4; 44.8	2.3; 28.6
*Age [%]*			
15	0.1; 1.4	0.0; 0.6	0.0; 0.3
16–45	1.9; 44.1	0.1; 19.1	0.9; 10.2
46–60	2.1; 18.2	0.1; 10.2	0.5; 6.3
61–75	4.3; 13.0	0.0; 8.7	0.4; 6.7
> 75	7.1; 8.4	0.1; 6.0	0.5; 5.0
Unknown	0.0; 0.2	0.0; 0.1	0.0; 0.1
*Sex [%]*			
Female	5.7; 26.8	0.1; 12.5	0.7; 7.9
Male	9.7; 58.6	0.3; 32.2	1.6; 20.7
*Region [%]*			
Central	1.9; 11.3	0.1; 6.0	0.4; 3.8
Northern	1.1; 5.8	0.0; 3.0	0.2; 1.9
Southeast	9.3; 53.7	0.1; 28.1	1.2; 17.9
Western	3.3; 14.6	0.1; 7.7	0.5; 5.0
*30-day mortality [% (n)]*			
Dead	17.6 (128); 1.5 (343)	0.0 (0); 1.9 (278)	13.1 (13); 86.9 (86)
Alive	82.4 (600); 98.5 (22,833)	100.0 (18); 98.1 (14,218)	2.5 (220); 97.5 (8,505)

Under- and overtriage rates presented as percentages and reported for (a) NTR, (b) the relative complement with OSISP-U5, and (c) the relative complement with OSISP-O35. Mortality, reported as percentage of patients and number in parenthesis, is presented for the under- and overtriage cohorts separately, divided into the subgroups dead and alive that combined sums up to 100%

The included data showed a mortality of 4.1% (*n* = 1,232 / 29,709). The potential reduction in mortality following the survival benefit analysis is presented in Table 10. For benefitted patients, OSISP had the estimated potential to save 203–223 lives, corresponding to a reduced mortality to 3.4% (*n* = 1,009 / 29,709) for OSISP-U5 and 3.5% (*n* = 1,029 / 29,709) for OSISP-O35. Corresponding results to Table 10 for the sensitivity analysis of alternative outcome definitions are presented in an additional file [see Additional file 3].

**Table 10:** Survival benefit estimation prior and posterior to OSISP intervention

Triage tool	Benefitted patients [n]	Prior intervention mortality [n]	Posterior intervention mortality [n]
OSISP-U5	4,001	447	224
OSISP-O35	3,464	407	204

## Discussion

### Summary of results

In this study we have conducted an external validation to evaluate the performance of the Swedish trauma model OSISP on unseen patient data from Norway. The test of independence confirmed a structured difference between the SweTrau and NTR registry data, justifying the latter as an appropriate source for assessing OSISP’s performance on future patients. OSISP applied on NTR data resulted in a successful validation of its overall ability to discriminate between severely injured and not severely injured, with similar performance as in the development study [26], and an overall alignment between predictions and observed outcomes with a mild overestimated risk for patients with a higher predicted risk of being severely injured. We observed suboptimal triage accuracy in current clinical outcome both according to ACS-COT and TTA definitions, with undertriage rates of 56.0% and 15.5%, respectively. For the clinical impact, OSISP as an independent CDSS has potential to improve undertriage substantially (from 56.0% to 5.0%) at the expense of increased overtriage (from 30.6% to 61.0%), while OSISP as a complementary CDSS improved both undertriage (from 56.0% to 2.7%) and overtriage (from 30.6% to 19.7%) so that ACS-COT’s recommended triage levels were reached. A similar trend on mortality impact was observed, with potential to reduce the overall mortality from 4.1% to 3.4–3.5% as a complementary CDSS. For vulnerable groups often incorrectly triaged in current clinical practice, such as elderly and patients from the northern region, we noted that OSISP has potential to reduce undertriage from 13.1% and 4.6% to between 0.2–1.7% and 0.3–1.0%, respectively. Overall, OSISP shows large potential to improve triage accuracy, which could lower mortality and improve medical outcome for trauma patients in Norway.

### Interpretation

#### Comparison of SweTrau and NTR registry data and current clinical triage accuracy

Several differences were found between the SweTrau and NTR registry data and their under- and overtriage rates. A larger proportion of data were excluded from NTR compared to SweTrau (Fig. 3). A control showed that 79.1% of the excluded NTR data were removed due to eligibility criteria (i.e., no involvement of prehospital resources, no direct transportation from the incident scene to the hospital, no adult trauma, or a prehospital death at the scene), which was considered a reasonable ground. The patient selection flowchart shows that the included NTR data have larger proportions of severely injured patients and smaller proportions of not severely injured patients compared to the included SweTrau data (Fig. 3). Also, the test of independence to compare predictors and outcomes between the registries found all but two predictors and all outcomes statistically different. Both SweTrau and NTR had high under- and overtriage rates (Table 4), independent of ACS-COT or TTA definitions. When comparing the registry data, a higher undertriage and lower overtriage were observed in NTR for ACS-COT calculations, and both a smaller under- and overtriage were observed in NTR for TTA calculations.

Combined, these results indicate that the NTR cohort is different compared to SweTrau’s, which is considered reasonable. Although both registries focus on major trauma and are based on the recommended variable setup from the Utstein protocol, four inclusion criteria are not shared (Table 1). This may cause NTR to include a broader trauma cohort compared to SweTrau. For the ACS-COT calculations, the higher undertriage and lower overtriage in NTR are expected, as NTR has fewer TCs compared to Sweden (four versus seven), making it more challenging to transport severely injured directly to TCs in Norway. Furthermore, the nature in Norway (e.g., fjords and high mountains) may also pose more challenging conditions to reach and transport patients [57], possibly causing severely injured patients to be transported to NTCs for later transfer to TCs. For the TTA calculations, the lower under- and overtriage in NTR should be interpreted carefully, as SweTrau’s TTA variable had a large degree of missing or unknown data (46.5%) for which the triage could not be calculated. The differences need to be considered when evaluating OSISP’s performance when applied on NTR data.

#### Overall model performance

It is common to observe a change in model performance compared to the development study when applying a model on external data [58]. The AUC_ROC_ and AUC_PR_ are two metrics to describe how well the model can differentiate between two classes [52, 53], i.e., between severely injured and not severely injured for OSISP. In the development study, OSISP trained to predict NISS > 15 and applied on the SweTrau data achieved an AUC_ROC_ of 0.88 and an AUC_PR_ of 0.62 [26]. In this study, OSISP applied on NTR data showed a small decrease in AUC_ROC_ of 0.83 and a small increase in AUC_PR_ of 0.64 (Table 5). The sensitivity analysis in this study also showed similar performance across the alternative outcome definitions for the AUC_ROC_ (0.82–0.86) and AUC_PR_ (0.65–0.68) except for the outcome ISS > 15 which had a reduced AUC_PR_ of 0.57 (Additional file 3). The decrease in AUC_ROC_ is considered small and may be explained by the differences between the SweTrau and NTR data. The increase of the AUC_PR_ is reasonable as NTR had a higher prevalence of severely injured. This enables a larger fraction of the predictions to be true positives and a smaller fraction to be false positives, which may reduce the denominator in the precision equation (Table 2) and thereby increase precision. The large drop in AUC_PR_ for ISS > 15 is therefore somewhat unexpected but aligns with the outcome sensitivity analysis in the development study [26]. A hypothesis is that the outcome ISS > 15 may not provide as clear patterns in the data as the other outcome definitions. Thus, the overall performance of OSISP is considered successfully validated for the outcomes ISS > 12, NISS > 12 and NISS > 15.

The calibration plot and metrics provide insight in how well predictions align with observed events [53]. Because OSISP trained to predict NISS > 15 produced a calibration curve that follows the diagonal line well (Fig. 4), the model is considered reasonably calibrated. Yet, there is a larger deviation for patients with a predicted risk of being severely injured above 0.6, and the small miscalibration is reflected in the Brier score, calibration slope and calibration in the large (Table 5). The consequence is an increased risk of false positives for patients with a predicted probability of being severely injured above 0.6, which may not necessarily be interpreted as a problem, but rather a safety net that supports the goal of reducing the undertriage. Whether OSISP’s miscalibration is acceptable or not will therefore depend on the healthcare organization’s goal for under- and overtriage. The sensitivity analysis of the outcomes resulted in similar calibration plots and interpretations supporting the conclusion of sufficient calibration, except for OSISP trained to predict NISS > 12 which in addition had larger deviations from the diagonal line within the probability interval about 0.3–0.5 (Additional file 3). As the calibration curve for the model trained to predict NISS > 12 is above the diagonal line, it may underestimate these patients, increasing the risk of false negatives and consequently increasing the risk of undertriage. As undertriage is often prioritized in trauma, recalibration or revising the model development for NISS > 12 is recommended.

#### Model performance at selected triage ability

Studying OSISP at the recommended field triage levels of 5% undertriage (OSISP-U5) and 35% overtriage (OSISP-O35) enables an estimation of its potential performance if deployed in clinical practice. Based on the different triage abilities and the imbalance of much fewer severely injured compared to not severely injured in the NTR data, differences in the proportions of true positives, true negatives, false positives, and false negatives were expected, primarily for the accuracy and PPV to be smaller for OSISP-U5 compared to OSISP-O35. This was confirmed in the results, as the F1-score and NPV were found comparable while the accuracy, PPV and recall had a moderate to large difference (Table 6). Similar trends could be seen in the sensitivity analysis of the alternative outcome definitions (Additional file 3). Clinically, this means that OSISP-U5 will be better at identifying severely injured as it has a higher recall (lower undertriage), while OSISP-O35 will have a more balanced ability to differentiate between severely injured and not severely injured. In clinical settings, low undertriage is often prioritized before a low overtriage, the healthcare organization must therefore decide what trade-off between undertriage and overtriage is acceptable. The development study did not report these metrics at a selected triage ability and could therefore not be used to compare the results.

The O/E ratio was low for both OSISP-U5 and OSISP-O35 (Table 6), and similar results were found in the sensitivity analysis of the alternative outcome definitions (Additional file 3). As this metric reflects the alignment between observed and predicted outcomes, the results indicate that both models overestimate the risk of a patient being severely injured. Clinically, this translates to reducing undertriage at the expense of overtriage and recalibration of the models may be warranted depending on the healthcare organisations’ acceptable levels for under- and overtriage.

#### Clinical impact at selected triage ability

OSISP as an independent CDSS means that only the predictions made by OSISP are used to triage patients. For this use case, both OSISP-U5 and OSISP-O35 demonstrated potential to reduce the undertriage from 56% to 5% and 16.8%, respectively. This can be considered a substantial and clinically important potential reduction. That major improvement in reducing undertriage came at the expense of an increased overtriage from 30.6% to 61% and 35%, respectively (Table 7). The extracted subgroups revealed similar trends of reduced undertriage at the expense of increased overtriage, except for the OSISP-O35 model for patients of 15–45 years of age and incidents in the central or western region where equal or reduced overtriage also could be observed. Similar results were found for the sensitivity analysis of alternative outcome definitions (Additional file 3).

OSISP as a complementary CDSS means that the triaging with current tools registered in NTR were combined with OSISP predictions to triage patients. For this use case, clinical impact was noted when basing the calculation of NTR’s triage on ACS-COT and TTA definitions. In the case of NTR triage based on ACS-COT definitions combined with OSISP’s predictions (Table 8), OSISP-U5 helped to reduce undertriage from 56% to 2.7% and overtriage from 30.6% to 19.7%, fulfilling ACS-COT’s recommended triage levels. Notably, the maximum under- and overtriage observed in the subgroups were 1.7% (males and the southeast region) and 13.8% (males), far below ACS-COT’s recommended triage levels. The OSISP-O35 also had a positive clinical impact and helped to reduce undertriage from 56% to 9.8% and overtriage from 30.6% to 11.8%, but not enough to fulfil ACS-COT’s recommended triage levels. Similar results were found for the sensitivity analysis of alternative outcome definitions (Additional file 3). In the case of NTR triage based on TTA definitions combined with OSISP’s predictions (Table 9), OSISP-U5 helped to reduce undertriage from 15.5% to 0.4% and overtriage from 85.4% to 44.8%, and OSISP-O35 helped to reduce undertriage from 15.5% to 2.3% and overtriage from 85.4% to 28.6%. For both models, all extracted subgroups had both improved under- and overtriage, with males being the subgroup most often incorrectly triaged. Because the recommended triage levels of 5% undertriage and 35% overtriage are defined for the ACS-COT definitions, comparison with these metrics were not considered appropriate.

Clinically, the results indicate that OSISP used as an independent CDSS has a theoretical potential to reduce undertriage, but not below ACS-COT’s recommended levels. However, OSISP evaluated at a 5% undertriage ability and used as a complementary tool has the theoretical potential to reduce both under- and overtriage below the recommended ACS-COT levels, as well as improved TTA triage, promoting further development of this particular use. From the literature, an increased risk for elderly to be incorrectly triaged has been found [16], and an increased risk of mortality has been reported for the northern region in Norway [17]. Based on the results for OSISP both as an independent and complementary CDSS, the undertriage in both groups could be improved (Tables 7, 8 and 9). The triage accuracy in each subgroup may further be used to complement the user guide on OSISP and alert the EMS personnel to be more observant for groups with a higher proportion of incorrect triage, for instance males or patients from the southeast region.

Improved triage ability can lead to reduced mortality by increasing the amount of severely injured patients who will receive adequate care at TC. OSISP as an independent tool reduced mortality in the undertriage group and increased mortality in the overtriage group (Table 7), while OSISP as a complementary tool reduced mortality in both the under- and overtriage groups (Tables 8 and 9). These results indicate that OSISP has the ability not only to improve the field triage performance, but also to detect patients in critical states. Yet, it is important to remember that the mortality analyses are based on retrospective data. In the clinical setting, multiple factors impact the patient outcome and improved field triage performance may not guarantee changes in mortality. The survival benefit analysis (Table 10) provides an alternative approach to assess the impact on mortality, where the estimated reduction of deaths following an intervention in the care process by using OSISP was 0.6–0.7 percentage points (*n* = 203–223). Considering that the NTR data used in the analysis represented eight years, OSISP may potentially save 25–27 lives per year in Norway. In addition to improved mortality, improved medical outcomes would also be expected. Similar results were found for the sensitivity analysis of alternative outcome definitions (Additional file 3). Overall, there are clear indications that OSISP can contribute to decreased mortality by increasing the proportion of patients who receive TC care, but prospective testing and investigations of preventable deaths are needed to validate the results.

### Comparison to existing models

Earlier versions of OSISP were developed on data from motor vehicle crashes. For Swedish truck occupant data, OSISP was trained to predict ISS > 15 and achieved an AUC_ROC_ of 0.81 for light trucks and 0.74 for heavy trucks [23]. Evaluation at an undertriage of 5% resulted in an overtriage of 85% for light trucks and 83% for heavy trucks [23]. For Swedish car occupant data, OSISP was trained to predict ISS > 8 and ISS > 15, achieving AUC_ROC_ of 0.78 and 0.83 [25]. When evaluated at an undertriage level of 10%, the models had an overtriage of 57% (ISS > 8) and 53% (ISS > 15) [25]. For US motor vehicle crash occupant data, OSISP was trained to predict ISS > 15 and achieved an AUC_ROC_ within the interval 0.78–0.86 [24]. Evaluation at an undertriage of 5% resulted in an overtriage of 59%, an overtriage of 25% resulted in an undertriage of 13% and an overtriage of 35% resulted in an undertriage of 20% [24].

The model developed by van Rein et al. was trained on Dutch trauma data to predict ISS > 15 and achieved an AUC_ROC_ of 0.82 in the development data, and had a calibration in the large of 0.89 and an AUC_ROC_ of 0.83 in external validation data [27]. The model was not evaluated at ACS-COT’s recommended triage levels, but had an undertriage of 11% at an overtriage of 50% [27]. In another external validation study of the Dutch model on data from England, the model had an AUC_ROC_ of 0.75, an O/E ratio of 2.47 and a calibration in the large of -1.02 [55]. After recalibration, the model had an AUC_ROC_ of 0.75, an O/E ratio of 1.0 and calibration in the large of 0.0 [55]. The model was not evaluated at ACS-COT’s recommended triage levels, but had an undertriage of 17% when evaluated at an overtriage of 50% [55]. In a subgroup analysis, the model had an undertriage of 12% when evaluated at the same overtriage rate [55].

In comparison to previous OSISP models and the Dutch model, OSISP in this study had a comparable AUC_ROC_ (0.83), except for the Dutch model validated on English data where OSISP performed better. The OSISP in this study improved overtriage compared to OSISP for truck incidents at 5% of undertriage, worsened the overtriage compared to OSISP on US data at 5% of undertriage, and improved undertriage compared to OSISP on US data at 35% of overtriage. The under- and overtriage following the OSISP model in this study could not be compared to the OSISP on motor vehicle crashes or the Dutch model due to different evaluation points. The observed differences in performance may be explained by previous OSISP models being developed on data representing a subset of injury mechanisms (motor vehicle crashes), while the OSISP model in this study is developed on all injury mechanisms registered by SweTrau.

### Limitations

Some variable levels were rare in the data (frequency < 20). Resampling the data during the development to even the distributions between predictor levels or grouping of predictor levels with few registrations may be a more appropriate method and ensure the model has utilized all data.

There is a lack of prehospital trauma data for the whole trauma population. Both SweTrau and NTR have good data quality [41, 59], however, both registries focus on collecting data from incidents that could represent major trauma. When reviewing the whole trauma population encountered by EMS personnel, an added group of less severely injured will be added, which OSISP has only seen a small fraction of during training and evaluation. For instance, Larsson et al. showed that during one year, 14% of the EMS missions in Sweden were related to trauma, of which less than 5% were registered as severely injured [60]. Similar trends would be expected for Norway. Training OSISP on data representing the whole trauma population would be beneficial, but such databases are not yet either available or matured.

This study evaluates OSISP on retrospective data and the interpretation of clinical impact is theoretical and must be done with care, since the triage calculations were simplified due to the shortcomings of not knowing the EMS personnel’s triage decision and the geographical location of the incident. Furthermore, hitherto we have not evaluated the ability of EMS personnel to use a tool such as OSISP in the field, and there may be practical and theoretical difficulties that could impede the real-world use and potential to improve triage. For instance, recall the simplifications made to calculate the triage for OSISP’s and the NTR registry data, e.g., that all patients predicted by OSISP to have a NISS > 15 will be considered transported to one of the four TCs in Norway and therefore be correctly triaged, while patients taken to NTCs with NISS > 15 in the NTR registry data will be considered undertriaged and be incorrectly triaged. These simplifications made in the present study will hinder confident conclusions on the clinical impact, as there may be local policies for EMS personnel to transport severely injured to hospitals not corresponding to TC, or the distance or transportation time may be too long. If the EMS personnel followed such policies those NTR registrations will be marked as incorrect when using the triage definitions in this study, which may result in pessimistically triaged registry data and optimistically triaged model data. Prospective evaluation of the model’s performance and clinical impact must therefore be conducted to validate the results.

### Future work

The OSISP model used in this study did not achieve a calibration in the large equal to zero. Recalibration of the model could therefore be needed. Furthermore, resampling the development data to improve the predictions on the identified under- and overtriage groups may be interesting to explore. However, such customization may reduce generalisability and cause overfitting on Norwegian data.

Further clinical investigation on how OSISP impacts additional patient-centered outcomes is recommended. For instance, information on intensive care unit admission, emergency surgery, massive transfusion, length of hospital stay, critical interventions and any permanent disability can help provide a clearer picture on preventable deaths and if the improved triage abilities translate to actual improvement in patient outcomes. Such analysis requires collection of related clinical data – from incident onset through prehospital and hospital care, to follow-up – and careful examination by clinical and statistical experts for each case of incorrectly triaged patients. It would also be of interest to study what specific injury types that benefit from TC care.

Most trauma research is observational, retrospective, not robust, and of low evidence [61]. Therefore, studies moving past the initial phases of model development is needed to improve the level of evidence. A suggestion for future work, based on the indicated clinical benefit, is therefore to move towards prospective evaluation of OSISP. For instance, digital platforms delivered by companies like Bliksund and Omda are used by EMS personnel in Norway. By integrating OSISP into such platforms, it can be tested prospectively to evaluate the clinical effectiveness. However, prior to such study, selecting which model (point on ROC curve) to test must be carefully evaluated in terms of possible impact in patients being delivered to TC and NTC, and calibrate it to the setting. Furthermore, a graphical user interface enabling efficient human-AI collaboration must be developed and evaluated by EMS personnel to ensure OSISP as a CDSS is usable.

## Conclusion

The OSISP model developed on Swedish trauma data to predict the risk of a patient being severely injured at the incident site was applied on external Norwegian trauma data to estimate its performance for future, unseen patients. The model’s ability to discriminate between severely injured and not severely injured was successfully validated, however, recalibration to better align predictions with observed outcomes may be needed depending on the healthcare organisation’s acceptable levels for under- and overtriage.

Substantial clinical benefits were observed with OSISP when applied on NTR data. As an independent CDSS, OSISP may substantially reduce undertriage. As a complementary CDSS in conjunction with current triage protocols, OSISP may substantially reduce both under- and overtriage, independent if using ACS-COT or TTA based triage calculations. In particular, OSISP shows a theoretical potential to improve triaging of groups often incorrectly triaged in current clinical practice, such as elderly and patients from the northern region in Norway, which contributes to an improved ability for healthcare organizations to provide equal care for all. Furthermore, OSISP as a complementary CDSS demonstrated potential to reduce mortality. In summary, using OSISP as a stand-alone or complementary CDSS shows large potential to improve triage accuracy, both in general and for vulnerable groups, which could lower mortality and improve medical outcome for trauma patients in Norway.

Based on the validated theoretical performance and potential clinical benefit, this study promotes further development of OSISP to be utilized as a complementary CDSS in conjunction with current triage protocols to support EMS personnel in the field triage of patients. Future research efforts should focus on activities enabling validation of the theoretical performance and clinical benefit in prospective studies, such as developing a graphical user interface to communicate the predictions and integrating the model into existing prehospital IT platforms.

## Supplementary Information

Below is the link to the electronic supplementary material.

Supplementary Material 1

Supplementary Material 2

Supplementary Material 3

## Data Availability

The raw data used in this study are available from the Swedish Trauma Registry (SweTrau) and the Norwegian Trauma Registry (NTR), but restrictions apply to the availability of these data, which were used under license for the current study and are not publicly available. For information about SweTrau, NTR and access to data, see their webpages https://rcsyd.se/swetrau/ and https://nkt-traume.no/nasjonalt-traumeregister/. For questions about requesting data from this study, contact the corresponding author, A.B.

## Abbreviations

ACS-COT: American College of Surgeons Committee on Trauma
AI: Artificial Intelligence
AIS: Abbreviated Injury Scale
AUC_PR_: Area Under Precision-Recall curve
AUC_ROC_: Area Under Receiver Operating Characteristic curve
CDSS: Clinical Decision Support System
ED: Emergency Department
EMS: Emergency Medical Services
FN: False Negative
FP: False Positive
ISS: Injury Severity Score
NISS: New Injury Severity Score
NPV: Negative Predictive Value
NTC: Non-Trauma Center
NTR: Norwegian Trauma Registry
O/E ratio: Observed/Expected ratio
OSISP: On-Scene Injury Severity Prediction
PPV: Positive Predictive Value
PR curve: Precision-Recall curve
ROC curve: Receiver Operating Characteristic curve
STRADA: Swedish Traffic Accident Data Acquisition
SweTrau: Swedish Trauma Registry
TC: Trauma Center
TN: True Negative
TP: True Positive
TRIPOD: Transparent Reporting of a multivariable prediction model for Individual Prognosis Or Diagnosis
TTA: Trauma Team Activation
XGBoost: Extreme Gradient Boosting
