# Supplementary material for: Evaluating performance and potential clinical benefit of the Swedish On Scene Injury Severity Prediction (OSISP) model for prehospital field triage on Norwegian trauma data
Source: Scand J Trauma Resusc Emerg Med. 2026 Jul 24;34:129. doi: 10.1186/s13049-026-01662-w (PMC13401289; doi:10.1186/s13049-026-01662-w)
Supplement: Supplementary file 2 — Supplementary Material 2 [file 13049_2026_1662_MOESM2_ESM.pdf]

Additional file 2. Software versions

Table S1. List of software versions used for the analysis.

**Programming language and packages**	**Version**
Python	3.8.5
openpyxl	3.1.2
xlrd	2.0.1
tzlocal	5.0.1
Pandas	1.5.3
Numpy	1.23.5
Scikit-learn (OneHotEncoder, calibration_curve, metrics, LogisticRegression)	1.2.2
SciPy (chi2_contingency, fisher_exact, logit)	1.9.3
XGBoost (XGBClassifier)	1.7.4
Matplotlib	3.7.1
R	4.2.0
rpy2	3.5.11
r-dplyr	1.1.2
stats (fisher_test)	4.2.0
