# Supplementary material for: Evaluating performance and potential clinical benefit of the Swedish On Scene Injury Severity Prediction (OSISP) model for prehospital field triage on Norwegian trauma data
Source: Scand J Trauma Resusc Emerg Med. 2026 Jul 24;34:129. doi: 10.1186/s13049-026-01662-w (PMC13401289; doi:10.1186/s13049-026-01662-w)
Supplement: Supplementary file 3 — Supplementary Material 3 [file 13049_2026_1662_MOESM3_ESM.pdf]

Additional file 3. Sensitivity analysis of outcome definition

This file presents results from the sensitivity analysis of the outcome on NTR data, with three alternative definitions of severely injured: ISS>12, ISS>15, and NISS>12.

Overall model performance on NTR data

Table S2 presents the overall OSISP performance (AUC_{ROC}, AUC_{PR}, Brier score, Calibration slope and Calibration in the large) when trained to the predict the alternative outcome definitions.

Table S2. Overall OSISP performance for alternative outcome definitions.

**Metric**	**ISS>12**	**ISS>15**	**NISS>12**
AUC_{ROC}	0.86	0.85	0.82
AUC_{PR}	0.65	0.57	0.68
Brier score	0.12	0.10	0.16
Calibration slope	0.84	0.81	0.81
Calibration in the large	-0.03	-0.12	0.07

AUC_{ROC} = Area under the Receiver Operating Characteristics curve, AUC_{PR} = Area under the Precision-Recall curve.

Figure S1–S3 present visualizations of the overall OSISP performance (ROC curve, PR curve and calibration curve) when trained to predict the alternative outcome definitions.

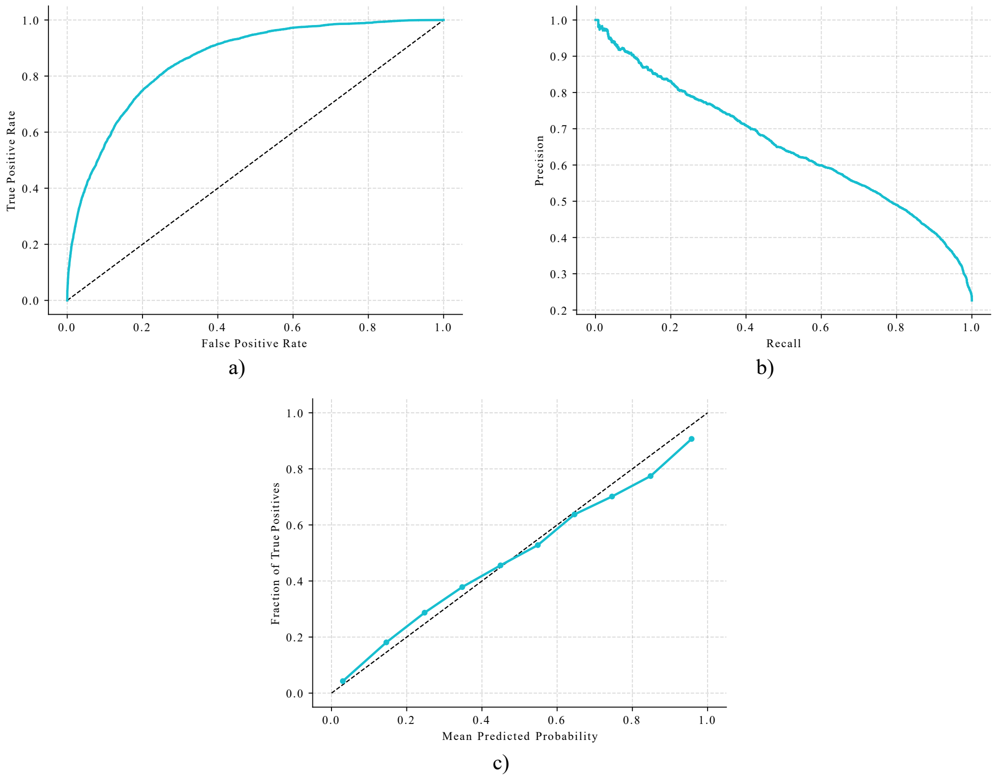

Figure S1. Overall performance visualizations of OSISP when trained to predict the outcome ISS>12. a) ROC curve, b) PR curve, c) calibration curve.

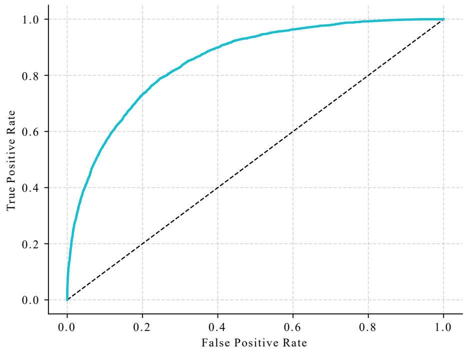

a)

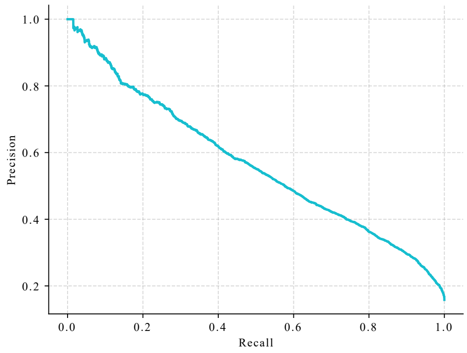

b)

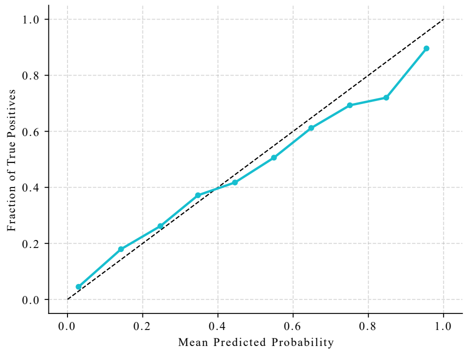

c)

Figure S2. Overall performance visualizations of OSISP when trained to predict the outcome ISS>15. a) ROC curve, b) PR curve, c) calibration curve.

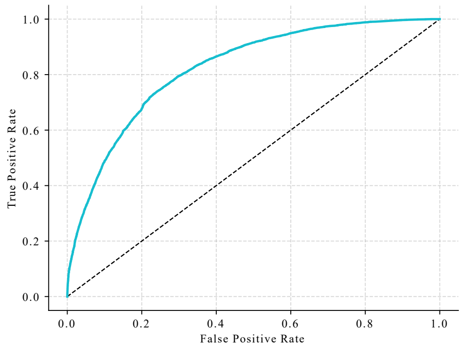

a)

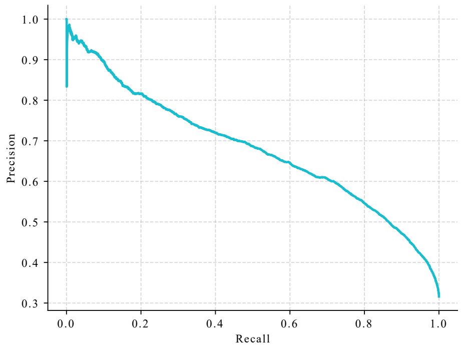

b)

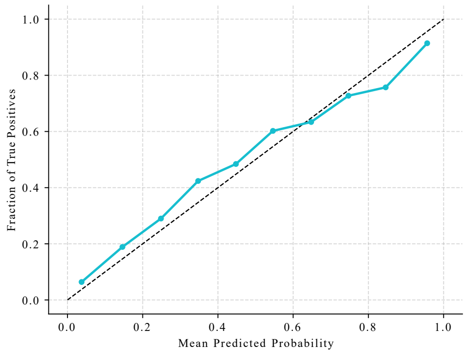

c)

Figure S3.Overall performance visualizations of OSISP when trained to predict the outcome NISS>12. a) ROC curve, b) PR curve, c) calibration curve.

Model evaluation at selected field triage ability: point performance on NTR data

Table S3 presents the OSISP point performance at the selected triage abilities (an undertriage of 5%, denoted as OSISP-U5, and an overtriage of 35% denoted as OSISP-O35) when trained to predict the alternative outcome definitions.

Table S3. OSISP performance evaluated at selected triage abilities.

	**OSISP-U5**			**OSISP-O35**		
**Metric**	ISS>12	ISS>15	NISS>12	ISS>12	ISS>15	NISS>12
Accuracy	0.60	0.54	0.57	0.70	0.68	0.71
F1-score	0.52	0.40	0.58	0.57	0.47	0.64
PPV	0.36	0.25	0.42	0.43	0.32	0.52
NPV	0.97	0.98	0.95	0.95	0.96	0.89
O/E ratio	0.37	0.26	0.44	0.48	0.37	0.63
Recall	0.95	0.95	0.95	0.88	0.87	0.83

PPV = Positive predictive value, NPV = Negative predictive value, O/E ratio = Observed/Expected ratio.

Evaluation of clinical impact: model as an independent CDSS

Table S4–S6 present the under- and overtriage in the NTR registry followed by the under- and overtriage by OSISP at the selected triage abilities (an undertriage of 5%, denoted as OSISP-U5, and an overtriage of 35% denoted as OSISP-O35). The triaged registry data were calculated with both ACS-COT and TTA definitions. The triaged OSISP data were calculated with ACS-COT definitions. Each table represent OSISP trained to predict each of the alternative outcome definitions.

Table S4. Under- and overtriage with OSISP (ISS>12) as independent CDSS.

	**NTR**[a]<br>Undertriage;<br>Overtriage	**OSISP-U5**[b]<br>Undertriage;<br>Overtriage	**OSISP-O35**[c]<br>Undertriage;<br>Overtriage
Total *[%]*	56.6; 31.2	5.0; 50.5	19.5; 25.0
Age [%]			
15	0.4; 0.5	0.1; 0.7	0.3; 0.2
16–45	15.3; 16.2	2.4; 21.4	8.5; 8.1
46–60	12.8; 6.3	1.2; 10.9	4.8; 5.1
61–75	14.6; 4.5	0.8; 9.6	3.2; 6.0
>75	13.3; 3.7	0.5; 7.7	2.6; 5.4
Unknown	0.3; 0.0	0.0; 0.1	0.0; 0.1
Sex [%]			
Female	16.0; 9.9	1.7; 14.7	5.4; 7.4
Male	40.6; 21.3	3.3; 35.8	14.0; 17.6
Region [%]			
Central	4.5; 4.9	0.8; 6.7	2.2; 3.4
Northern	4.5; 1.8	0.5; 3.4	1.5; 1.7
Southeast	36.9; 18.1	2.9; 31.5	12.1; 15.4
Western	10.7; 6.4	0.9; 8.9	3.7; 4.4
30-day mortality [% (n)]			
Dead	12.0 (456);<br>1.8 (131)	13.7 (46);<br>2.2 (256)	9.9 (77);<br>2.9 (232)
Alive	88.0 (3,353);<br>98.2 (7,032)	86.3 (290);<br>97.8 (11,349)	90.1 (697);<br>97.1 (7,806)

Under- and overtriage rates presented as percentages and reported for a) NTR based on ACS-COT definitions, b) OSISP-U5 based on ACS-COT definitions, and c) OSISP-O35 based on ACS-COT definitions. Mortality, reported as percentage of patients and number in parenthesis, is presented for the under- and overtriage cohorts separately, divided into the subgroups dead and alive that combined sums up to 100%.

Table S5. Under- and overtriage with OSISP (ISS>15) as independent CDSS.

	**NTR**[a] Undertriage; Overtriage	**OSISP-U5**[b] Undertriage; Overtriage	**OSISP-O35**[c] Undertriage; Overtriage
Total *[%]*	53.3; 31.6	5.0; 53.6	20.9; 25.0
Age [%]			
15	0.4; 0.5	0.1; 0.7	0.3; 0.2
16–45	14.1; 15.8	2.6; 21.3	9.3; 7.2
46–60	11.1; 6.7	1.3; 11.9	5.2; 5.1
61–75	13.9; 4.7	0.6; 10.8	3.6; 6.4
>75	13.6; 3.9	0.4; 8.7	2.4; 6.0
Unknown	0.2; 0.0	0.0; 0.1	0.0; 0.0
Sex [%]			
Female	15.5; 9.8	1.6; 15.5	5.6; 7.6
Male	37.9; 21.7	3.4; 38.0	15.3; 17.4
Region [%]			
Central	3.8; 5.0	0.7; 7.1	2.3; 3.3
Northern	4.1; 1.8	0.4; 3.7	1.6; 1.7
Southeast	34.6; 18.4	3.1; 33.5	13.0; 15.6
Western	10.8; 6.4	0.8; 9.3	4.0; 4.3
30-day mortality [% (n)]			
Dead	16.6 (416); 1.8 (144)	15.0 (35); 2.4 (324)	10.5 (65); 3.2 (282)
Alive	83.4 (2,091); 98.2 (7,747)	85.0 (199); 97.6 (13,076)	89.5 (555); 96.8 (8,470)

Under- and overtriage rates presented as percentages and reported for a) NTR based on ACS-COT definitions, b) OSISP-U5 based on ACS-COT definitions, and c) OSISP-O35 based on ACS-COT definitions. Mortality, reported as percentage of patients and number in parenthesis, is presented for the under- and overtriage cohorts separately, divided into the subgroups dead and alive that combined sums up to 100%.

Table S6. Under- and overtriage with OSISP (NISS>12) as independent CDSS.

	**NTR**[a] Undertriage; Overtriage	**OSISP-U5**[b] Undertriage; Overtriage	**OSISP-O35**[c] Undertriage; Overtriage
Total *[%]*	58.1; 30.3	5.0; 60.3	25.3; 25.0
Age [%]			
15	0.4; 0.5	0.1; 1.0	0.3; 0.3
16–45	14.8; 16.7	3.0; 28.0	11.7; 8.6
46–60	12.4; 6.2	1.1; 12.9	6.0; 5.2
61–75	15.1; 4.0	0.6; 10.5	4.1; 5.7
>75	15.3; 2.9	0.3; 7.6	3.1; 5.1
Unknown	0.2; 0.0	0.0; 0.2	0.0; 0.1
Sex [%]			
Female	18.5; 9.5	2.1; 16.8	7.9; 7.0
Male	39.6; 20.8	2.9; 43.4	17.4; 18.0
Region [%]			
Central	5.0; 4.6	0.6; 8.2	3.0; 3.4
Northern	4.8; 1.8	0.4; 4.2	1.9; 1.8
Southeast	37.9; 17.2	3.2; 36.9	16.2; 15.4
Western	10.5; 6.6	0.8; 10.9	4.2; 4.5
30-day mortality [% (n)]			
Dead	9.8 (537); 1.4 (87)	12.4 (58); 1.7 (209)	5.9 (92); 2.5 (177)
Alive	90.2 (4,919); 98.6 (6,065)	87.6 (411); 98.3 (12,038)	94.1 (1,466); 97.5 (6,926)

Under- and overtriage rates presented as percentages and reported for a) NTR based on ACS-COT definitions, b) OSISP-U5 based on ACS-COT definitions, and c) OSISP-O35 based on ACS-COT definitions. Mortality, reported as percentage of patients and number in parenthesis, is presented for the under- and overtriage cohorts separately, divided into the subgroups dead and alive that combined sums up to 100%.

Evaluation of clinical impact: model as a complementary CDSS

Table S7–S9 present the under- and overtriage in the NTR registry followed by the relative complemented under- and overtriage by OSISP at the selected triage abilities (an undertriage of 5%, denoted as OSISP-U5, and an overtriage of 35% denoted as OSISP-O35). The triaged registry data were calculated with ACS-COT definitions, and the relative complement was calculated for OSISP trained to predict each of the alternative outcomes. Table S10 presents the survival benefit analysis for the alternative outcomes.

Table S7. Under- and overtriage with OSISP (ISS>12) as complementary CDSS for ACS-COT definitions.

	**NTR**[a] Undertriage; Overtriage	**NTR&OSISP-U5**[b] Undertriage; Overtriage	**NTR&OSISP-O35**[c] Undertriage; Overtriage
Total *[%]*	56.6; 31.2	2.7; 17.0	6.8; 12.2
Age [%]			
15	0.4; 0.5	0.0; 0.2	0.2; 0.1
16–45	15.3; 16.2	1.2; 7.7	2.8; 4.9
46–60	12.8; 6.3	0.7; 3.6	1.7; 2.5
61–75	14.6; 4.5	0.5; 3.0	1.1; 2.4
>75	13.3; 3.7	0.3; 2.5	1.0; 2.2
Unknown	0.3; 0.0	0.0; 0.0	0.0; 0.0
Sex [%]			
Female	16.0; 9.9	0.9; 4.9	2.0; 3.5
Male	40.6; 21.3	1.9; 12.1	4.8; 8.8
Region [%]			
Central	4.5; 4.9	0.3; 2.7	0.6; 1.9
Northern	4.5; 1.8	0.3; 1.0	0.6; 0.7
Southeast	36.9; 18.1	1.6; 10.3	4.3; 7.5
Western	10.7; 6.4	0.4; 3.1	1.2; 2.2
30-day mortality [% (n)]			
Dead	12.0 (456); 1.8 (131)	20.0 (37); 2.3 (90)	12.9 (59); 2.9 (81)
Alive	88.0 (3,353); 98.2 (7,032)	80.0 (148); 97.7 (3,825)	87.1 (399); 971 (2,727)

Under- and overtriage rates presented as percentages and reported for a) NTR, b) the relative complement with OSISP-U5, and c) the relative complement with OSISP-O35. Mortality, reported as percentage of patients and number in parenthesis, is presented for the under- and overtriage cohorts separately, divided into the subgroups dead and alive that combined sums up to 100%.

Table S8. Under- and overtriage with OSISP (ISS>15) as complementary CDSS for ACS-COT definitions.

	**NTR**[a] Undertriage; Overtriage	**NTR&OSISP-U5**[b] Undertriage; Overtriage	**NTR&OSISP-O35**[c] Undertriage; Overtriage
Total *[%]*	53.3; 31.6	2.7; 18.4	7.3; 12.4
Age [%]			
15	0.4; 0.5	0.1; 0.2	0.1; 0.1
16–45	14.1; 15.8	1.3; 7.9	3.5; 4.5
46–60	11.1; 6.7	0.7; 4.1	1.7; 2.6
61–75	13.9; 4.7	0.3; 3.3	1.0; 2.7
>75	13.6; 3.9	0.3; 2.9	1.0; 2.5
Unknown	0.2; 0.0	0.0; 0.0	0.0; 0.0
Sex [%]			
Female	15.5; 9.8	0.9; 5.3	1.9; 3.6
Male	37.9; 21.7	1.8; 13.1	5.4; 8.8
Region [%]			
Central	3.8; 5.0	0.3; 3.0	0.6; 2.0
Northern	4.1; 1.8	0.4; 1.0	0.7; 0.7
Southeast	34.6; 18.4	1.6; 11.2	4.4; 7.6
Western	10.8; 6.4	0.4; 3.2	1.5; 2.2
30-day mortality [% (n)]			
Dead	16.6 (416); 1.8 (144)	20.6 (26); 2.3 (107)	14.0 (48); 3.1 (97)
Alive	83.4 (2,091); 98.2 (7,747)	79.4 (100); 97.7 (4,494)	86.0 (296); 96.9 (3,008)

Under- and overtriage rates presented as percentages and reported for a) NTR, b) the relative complement with OSISP-U5, and c) the relative complement with OSISP-O35. Mortality, reported as percentage of patients and number in parenthesis, is presented for the under- and overtriage cohorts separately, divided into the subgroups dead and alive that combined sums up to 100%.

Table S9. Under- and overtriage with OSISP (NISS>12) as complementary CDSS for ACS-COT definitions.

	**NTR**[a] Undertriage; Overtriage	**NTR&OSISP-U5**[b] Undertriage; Overtriage	**NTR&OSISP-O35**[c] Undertriage; Overtriage
Total *[%]*	58.1; 30.3	2.9; 19.3	9.8; 11.5
Age [%]			
15	0.4; 0.5	0.1; 0.3	0.2; 0.1
16–45	14.8; 16.7	1.7; 9.6	4.6; 4.9
46–60	12.4; 6.2	0.7; 4.1	2.3; 2.4
61–75	15.1; 4.0	0.3; 3.0	1.4; 2.2
>75	15.3; 2.9	0.2; 2.4	1.3; 1.9
Unknown	0.2; 0.0	0.0; 0.0	0.0; 0.0
Sex [%]			
Female	18.5; 9.5	1.2; 5.4	3.3; 3.1
Male	39.6; 20.8	1.8; 13.9	6.4; 8.4
Region [%]			
Central	5.0; 4.6	0.2; 3.1	0.9; 1.8
Northern	4.8; 1.8	0.4; 1.3	1.0; 0.8
Southeast	37.9; 17.2	1.8; 10.9	6.4; 6.8
Western	10.5; 6.6	0.5; 4.0	1.4; 2.3
30-day mortality [% (n)]			
Dead	9.8 (537); 1.4 (87)	16.1 (44); 1.8 (72)	7.3 (67); 2.5 (58)
Alive	90.2 (4,919); 98.6 (6,065)	83.9 (230); 98.2 (3,842)	92.7 (852); 97.5 (2,289)

Under- and overtriage rates presented as percentages and reported for a) NTR, b) the relative complement with OSISP-U5, and c) the relative complement with OSISP-O35. Mortality, reported as percentage of patients and number in parenthesis, is presented for the under- and overtriage cohorts separately, divided into the subgroups dead and alive that combined sums up to 100%.

Table S10. Survival benefit estimation prior and posterior to OSISP intervention.

**Outcome**	**Triage tool**	**Benefitted patients** [n]	**Prior intervention mortality** [n]	**Posterior intervention mortality** [n]
ISS>12	OSISP-U5	3,624	419	210
	OSISP-O35	3,351	397	199
ISS>15	OSISP-U5	2,381	390	195
	OSISP-O35	2,163	368	184
NISS>12	OSISP-U5	5,182	493	247
	OSISP-O35	4,537	470	235

For ISS>15, OSISP-U5 and OSISP-O35 reduced mortality in the included data to 3.5% (n=1,037 / 29,709) and 3.5% (n=1,048 / 29,709), respectively. For ISS>12, OSISP-U5 and OSISP-O35 reduced mortality in the included data to 3.4% (n=1,023 / 29,709) and 3.5% (n=1,034 / 29,709), respectively. For NISS>12, OSISP-U5 and OSISP-O35 reduced mortality in the included data to 3.3% (n=986 / 29,709) and 3.4% (n=997 / 29,709), respectively.
